# Conversion of Substrate Analogs Suggests a Michael Cyclization in Iridoid Biosynthesis

**DOI:** 10.1016/j.chembiol.2014.09.010

**Published:** 2014-11-20

**Authors:** Stephanie Lindner, Fernando Geu-Flores, Stefan Bräse, Nathaniel H. Sherden, Sarah E. O’Connor

**Affiliations:** 1 Department of Biological Chemistry, The John Innes Centre, Norwich NR4 7UH, UK; 2 Institute of Organic Chemistry, Karlsruhe Institute of Technology, Fritz-Haber-Weg 6, 76131 Karlsruhe, Germany; 3 Institute of Toxicology and Genetics, Hermann-von-Helmholtz-Platz 1, 76344 Eggenstein-Leopoldshafen, Germany

## Abstract

The core structure of the iridoid monoterpenes is formed by a unique cyclization reaction. The enzyme that catalyzes this reaction, iridoid synthase, is mechanistically distinct from other terpene cyclases. Here we describe the synthesis of two substrate analogs to probe the mechanism of iridoid synthase. Enzymatic assay of these substrate analogs along with clues from the product profile of the native substrate strongly suggest that iridoid synthase utilizes a Michael reaction to achieve cyclization. This improved mechanistic understanding will facilitate the exploitation of the potential of iridoid synthase to synthesize new cyclic compounds from nonnatural substrates.

## Highlights

• Iridoid synthase can turn over substrate analogs
• Iridoid synthase yields both open and closed forms of its native product
• The combined evidence suggests that iridoid synthase uses a Michael reaction

## Graphical Abstract

**Figure:**
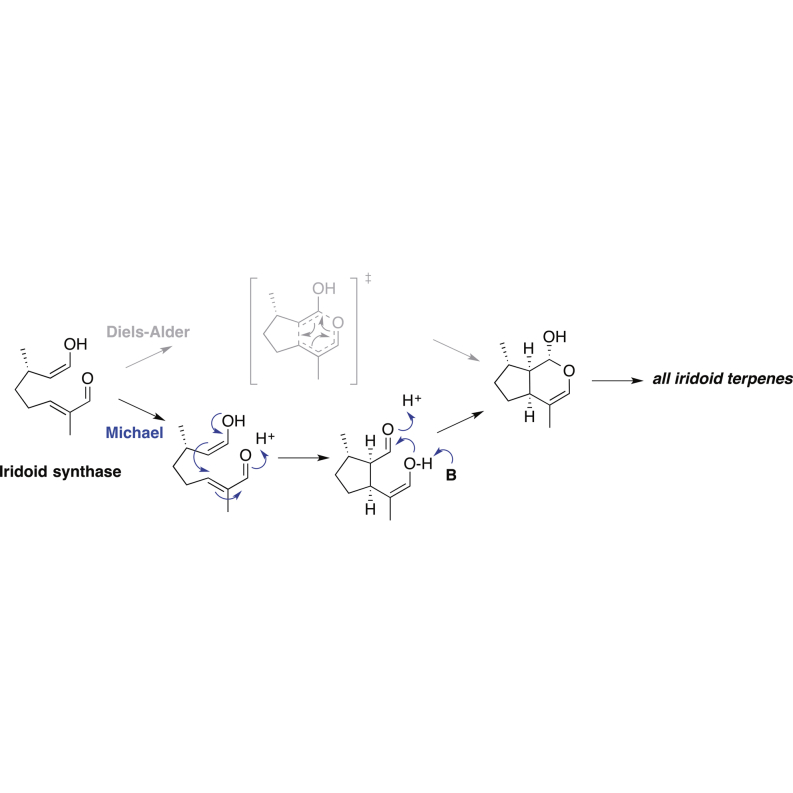

## Teaser

Enzymatic assay of two substrate analogs, along with clues from the product profile of the native substrate, strongly suggest that the iridoid synthase enzyme utilizes a Michael reaction to achieve cyclization.

## Introduction

**Figure 1:**
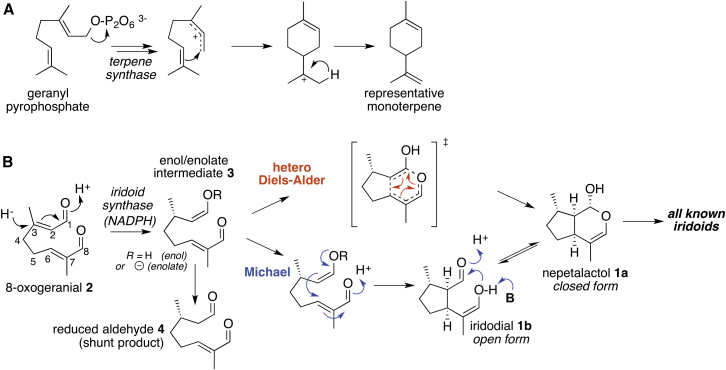
Terpene Cyclization (A) Representative canonical terpene cyclization mechanism. (B) Iridoid synthase uses 8-oxogeranial (**2**) as a substrate. A hydride from NADPH reduces the substrate to an enol or enolate **3**, which can then cyclize to **1a**. Two possible cyclization mechanisms are possible for iridoid synthase: a concerted hetero Diels-Alder (red arrows) and a stepwise Michael addition (blue arrows).

The iridoids are a distinct class of approximately 600 monoterpenes that display a broad range of pharmacological and agrochemical activities (Tundis et al., 2008, Dewhirst et al., 2010, Dinda et al., 2011). We recently reported the discovery of iridoid synthase, the enzyme that produces nepetalactol (**1a**), the common biosynthetic precursor for all iridoids (Geu-Flores et al., 2012). Notably, this enzyme is mechanistically distinct from canonical terpene synthases (Uesato et al., 1983, Uesato et al., 1984, Uesato et al., 1987). Instead of forming a reactive cationic species from geranyl pyrophosphate (Figure 1A) (Degenhardt et al., 2009, Chen et al., 2011, Kim et al., 2012), iridoid synthase catalyzes cyclization that is triggered by reduction of 8-oxogeranial (**2**) to form enol or enolate intermediate **3**. Intermediate **3** is poised to cyclize to form nepetalactol (**1a**) by either an inverse electron demand hetero Diels-Alder (for examples of enzymatic Diels-Alder reactions, see Kim et al., 2012) or a Michael reaction (for examples of enzymatic Michael reactions, see Kusebauch et al., 2009, Bretschneider et al., 2013) (Figure 1B). Here we describe the synthesis and enzymatic assay of two substrate analogs designed to probe which reaction pathway iridoid synthase favors for cyclization. On the basis of these studies, along with clues from the product profile of the native substrate, it appears that iridoid synthase utilizes a Michael addition reaction mechanism for cyclization of the iridoid class of natural products. This provides an essential piece of the mechanistic puzzle of how the iridoid scaffold is constructed.

## Results and Discussion

After iridoid synthase reduces 8-oxogeranial (**2**) using nicotinamide adenine dinucleotide phosphate (NADPH) as hydride (H^–^) donor, enol or enolate intermediate **3** is formed (Figure 1B). The existence of reaction intermediate **3** is supported by the identification of reduced aldehyde **4**, the more stable tautomer of **3**, as a minor product in the iridoid synthase catalyzed reaction (Geu-Flores et al., 2012). Moreover, the formation of an enol intermediate is entirely consistent with the proposed mechanism of progesterone-β-reductase (Thorn et al., 2008, Bauer et al., 2010), which displays high sequence similarity to iridoid synthase (67% amino acid identity compared with *Digitalis purpurea* P5bR2). Once formed, **3** can cyclize to form the characteristic bicyclic 5-6 ring iridoid framework of nepetalactol (**1a**). However, the specific mechanism of this cyclization is cryptic. In one scenario, cyclization could occur by a stepwise Michael reaction, forming the 5-membered ring first, with subsequent cyclization to the lactol (Figure 1B, blue arrows). Alternatively, the reaction could proceed via an inverse electron demand hetero Diels-Alder reaction (Figure 1B, red arrows).

**Figure 2:**
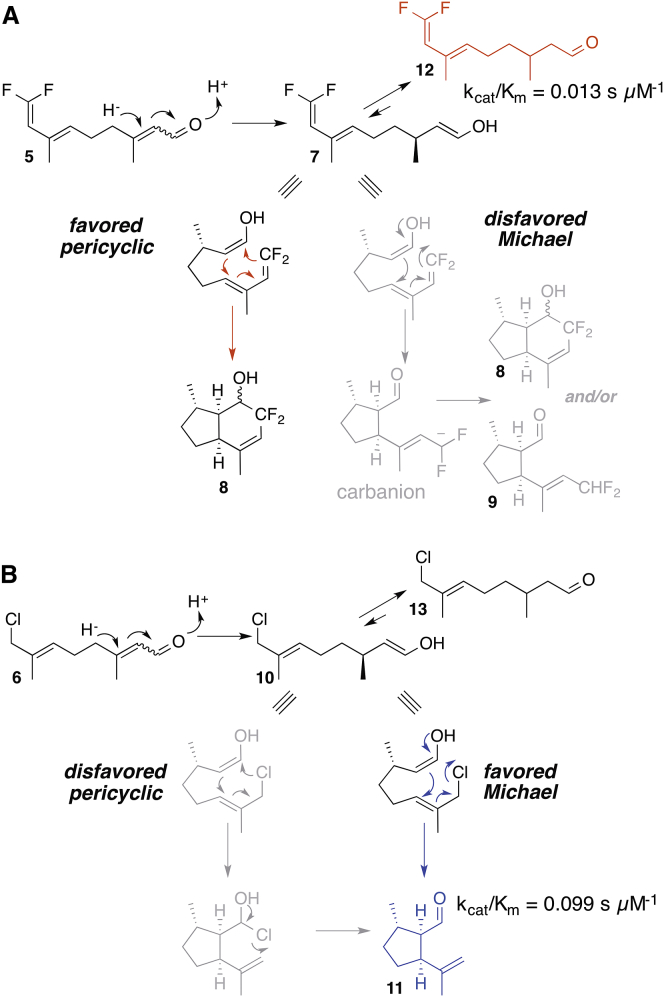
Substrate Analogs (A) Compound **5** is poised to undergo a Diels-Alder reaction (red arrows) upon enol (or enolate) formation, while the Michael reaction is disfavored. (B) Compound **6** is predisposed to undergo a Michael reaction (blue arrows) and is unlikely to undergo the Diels-Alder related Halo-Alder-ene pericyclic reaction. Disfavored mechanisms are shown in gray. Compounds **7** and **10** are shown in enol forms. Enzymatic products that were isolated are shown in red, along with corresponding *k*_cat_ and *K*_*M*_ values.

To distinguish between these two mechanistic possibilities, two substrate analogs theoretically capable of cyclization by iridoid synthase were synthesized. One substrate, compound **5**, was designed to disfavor the Michael mechanism while favoring a Diels-Alder reaction; the other, compound **6**, strongly disfavored the Diels-Alder reaction while favoring the Michael reaction (Figure 2). Provided that both can be accommodated within the enzyme active site, cyclization of only one of these substrates by the enzyme would suggest the more likely reaction mechanism for the native substrate.

Iridoid synthase is predicted to reduce compound **5** to enol/enolate intermediate **7**. Intermediate **7** harbors a diene with electron withdrawing groups (fluorine) and a dienophile with an electron donating group (OH or O^–^) and could therefore undergo an inverse electron demand Diels-Alder to form product **8** (Figure 2A); precedent for fluorinated dienes in enhancing Diels-Alder reactions exists (Kaz’mina et al., 1984, Roversi et al., 2002, Vogel et al., 2007). In contrast, the Michael addition with substrate **5** entails formation of a carbanion species (Figure 2A), which is far less stable than the enol or enolate species that would occur with the native substrate (Figure 1B). Although the carbanion could be somewhat stabilized because of the inductive electron-withdrawing effects of the fluorine atoms, this effect is greatly mitigated because of electron-pair repulsions between the carbanion and the fluorine lone pairs (Zhang et al., 2012). Altogether, compared with the native substrate **2**, **5** is a much weaker candidate for a Michael addition. Thus the formation of cyclization product **8** upon incubation of iridoid synthase with substrate **5** would suggest that the enzyme utilizes a pericyclic reaction mechanism.

Candidate **6** is intended to undergo conjugate reduction in the enzyme to form intermediate **10**, which is primed to perform an S_N_2′ conjugate addition to give cyclization product **11** (Figure 2B). It is highly unlikely for intermediate **10** to undergo a Diels-Alder equivalent reaction (a halo-Alder-ene); we have found no precedent for such a reaction. Therefore, formation of cyclized product **11** would suggest that the enzyme utilizes a Michael reaction mechanism.

**Figure 3:**
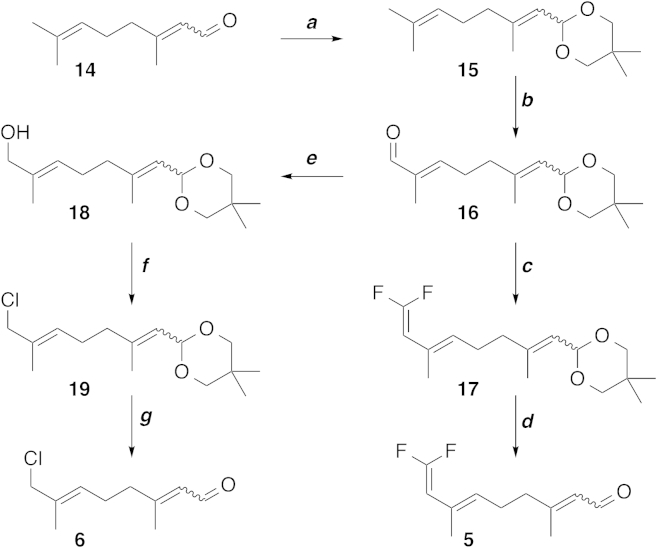
Synthetic Procedures for Compounds **5** and **6** (A) *p*-Toluenesulfonic acid monohydrate, 2,2-dimethylpropane-1,3-diol, benzene, 110°C, 2 hr (quant.) (B) SeO_2_, Na_2_SO_4_, dichloromethane, reaction time (r.t.), 2 d (11%). (C) Sodium chlorodifluoroacetate, PPh_3_, dimethylformamide, 100°C, 3 hr (18%). (D) HCl, THF, r.t., 1 hr (71%). (E) NaBH_4_, MeOH, 0°C-r.t., 1.5 hr (36%). (F) Tosyl chloride, 4-dimethylaminopyridine, NEt_3_, dichloromethane, r.t., 3 hr (45%). (G) Trifluoroacetic acid/H_2_O (1:1), dichloromethane, r.t., 10 min (69%). Full details are provided in the Supplemental Information.

The synthesis of both **5** and **6** started with the acetalization of citral (**14**), followed by the allylic oxidation of one methyl group using stoichiometric amounts of SeO_2_ to yield **16** (Figure 3; Supplemental Information available online). Ensuing difluoromethylenation with sodium chlorodifluoroacetate followed by hydrolysis of the acetal led to the Diels-Alder test substrate, 8-(difluoromethylene)geranial (**5**). The Michael test substrate, 8-chlorogeranial (**6**), was obtained from **16** by reduction of the aldehyde using sodium borohydride, chlorination with tosyl chloride followed by deacetalization (Figure 3; Supplemental Information). Compounds **5** and **6** were incubated with iridoid synthase and product formation was assessed by gas chromatography-mass spectrometry (GC-MS). The major products for both enzymatic reactions were also isolated, purified, and then characterized by nuclear magnetic resonance (NMR), further validating the structures of the enzymatic products (SI).

Upon incubation of iridoid synthase with substrate **5**, the linear reduction product **12** was observed (Figure 2A, red compound). This indicates that iridoid synthase is catalytically competent with **5**, despite the perturbations to the native substrate structure. Additionally, the lack of observable cyclized product **8** shows that the enzyme does not favor the Diels-Alder cyclization mechanism for which this substrate was designed. In contrast, when iridoid synthase was incubated with compound **6**, cyclized product **11** could be cleanly isolated (Figure 2B, blue compound). Given that a pericyclic reaction for compound **6** is highly disfavored, it seems most likely that the observed cyclization occurs via the Michael reaction. Nuclear Overhauser effect spectroscopy NMR spectra suggest that the product has the relative stereochemistry shown (Supplemental Information), which matches that of the native enzyme product **1b**.

Compounds **5** and **6** were subjected to steady-state kinetic analysis (Supplemental Information). Compound **5** (*K*_*M*_ = 485 ± 160 μM, *k*_cat_ = 6.4 ± 0.8 s^−1^, *k*_cat_/*K*_*M*_ = 0.013 s μM^−1^; Supplemental Information) had a catalytic efficiency 8-fold less than that observed for compound **6** (*K*_*M*_ = 81.9 ± 5.6 μM, *k*_cat_ = 8.1 ± 0.5 s^−1^, *k*_cat_/*K*_*M*_ = 0.099 s μM^−1^; Supplemental Information). Although both **5** and **6** had lower catalytic efficiencies than that observed for natural substrate **2** (*K*_*M*_ = 9.9 ± 2.1 μM, *k*_cat_ = 1.4 ± 0.1 s^−1^, *k*_cat_/*K*_*M*_ = 0.14 s^−1^ μM^−1^), the steady-state kinetic measurements confirm that both **5** and **6** are competent substrates, though only substrate **6** was cyclized. For these studies, an enzyme with a truncation at the N terminus was used, which increases the structural stability of the nearest iridoid synthase homolog, progesterone beta-reductase. This truncation has recently been shown to affect kinetic parameters for progesterone beta-reductase (Rudolph et al., 2014). Therefore, kinetic parameters for **2** were remeasured using this truncated enzyme. For comparison, kinetic parameters for the full-length enzyme with **2** are *K*_*M*_ = 4.5 ± 0.2 μM, *k*_cat_ = 1.6 ± 0.1 s^−1^, *k*_cat_/*K*_*M*_ = 0.36 s^−1^ μM^−1^.

The mechanistic implications drawn from substrate analogs must be interpreted with caution. For example, both analogs **5** and **6** had a higher *K*_*M*_ than native substrate **2**, but the difference was more marked for **5**, which might be indicative of an impaired binding to the active site. Additionally, the electronic properties of both analogs could be modulated by hydrogen bonding interactions with the enzyme, thereby altering the propensity of the compounds to cyclize via a Diels-Alder or Michael reaction. Ideally, comparison of nonenzymatic cyclization reactions with enzyme-catalyzed reactions would provide more insight into the baseline reactivity of these compounds. Unfortunately, we were unable to chemically cyclize these compounds. After chemical reduction of a more stable and synthetically accessible analog of **5** (9,9-difluoro-2,6-dimethylnona-2,6,8-trienal) using L-selectride to generate the reactive enol/enolate, we only obtained the alcohol (9,9-difluoro-2,6-dimethylnona-2,6,8-trien-1-ol). Reduction using Stryker’s reagent in combination with LiCl or TMSCl led to an unidentifiable product mixture. Efforts to generate a protected enol species that could be subjected to chemical conditions favorable for a Diels-Alder reaction were unsuccessful. Efforts to assess whether **6** could cyclize nonenzymatically were complicated by the propensity of **6** to rearrange in solution. Despite these caveats, the results from enzymatic assay with the two substrate analogs are consistent: substrate **5**, which is primed for a Diels-Alder reaction, failed to cyclize, whereas substrate **6**, primed for a Michael reaction, did cyclize. Therefore, it seems reasonable to conclude that the cyclization step of iridoid synthase likely operates via a Michael addition reaction mechanism.

Finally, it is prudent to consider whether the product distribution that results from native substrate **2** also supports this mechanism. The Michael reaction proceeds via the open form of nepetalactol (**1a**), iridodial (**1b**), while the Diels-Alder proceeds directly to the closed form **1a** (Figure 1B). The native iridoid synthase cyclization product appears as a mixture of the closed and open forms, **1a** and **1b**, as evidenced by GC-MS and TLC (Geu-Flores et al., 2012). We reported in our initial experiments that **1a** and **1b** are in equilibrium (Geu-Flores et al., 2012), which would mean that the presence of both the open and closed forms provides no insight into a mechanistic hypothesis. We have now performed a more detailed analysis of the product distribution of **1a** and **1b,** which demonstrates that the amount of open form observed is in fact greatly dependent upon the temperature of the GC inlet (low GC inlet temperatures have shown the stability of the open form), as well as on its usage history (Dawson et al., 1989). With this knowledge at hand, it is clear that the open and closed forms of **1** equilibrate on a much slower timescale than previously assumed (see the Supplemental Information for detailed information and data). Therefore, we can now conclude that both the open and closed forms are produced in the enzymatic reaction. Because the Diels-Alder mechanism does not involve the open form **1b**, we would be less likely to observe the open form if the enzyme used a pericyclic cyclization. The presence of both open and closed forms of **1** is also more consistent with a cyclization mechanism utilizing the Michael reaction. Although the slow equilibrium would suggest that the stereochemistry at the hemi-acetal carbon of the closed form **1a** could provide mechanistic insight into the nature of the cyclization reaction, epimerization can also occur via acid-catalyzed loss of lactolic OH to give an oxocarbenium intermediate. Therefore, we have not considered the stereochemistry of **1a** at this carbon as supportive of one mechanism over the other.

In synthetic systems, intramolecular cyclization of dicarbonyl substrates to form the iridoid scaffold has utilized both Diels-Alder and Michael addition mechanisms. For example, a domino Knoevenagel-hetero-Diels-Alder reaction has been employed to form the iridoid scaffold (Tietze and Bartels, 1991), and an enol ether derivative of a trialdehyde substrate also cyclized immediately via an intramolecular inverse electron demand Diels-Alder to yield an iridoid derivative (Tietze et al., 1980, Tietze et al., 1982, Tietze, 1983). However, intramolecular cyclization of dicarbonyl substrates to yield iridoids has also been achieved via Michael reaction using a Jørgensen-Hayashi catalyst (Marqués-López et al., 2009), and a reductive Michael cyclization of a keto aldehyde has been reported (Yang et al., 2005). Although the inherent chemical reactivity of the linear iridoid precursor is compatible with both reactions, our studies suggest that nature utilizes the Michael reaction.

Iridoid synthase joins a growing list of diverse enzymes that catalyze unusual terpene cyclization reactions (Itoh et al., 2010, Shoyama et al., 2012, Xu et al., 2012). Although substrate probes and product identities cannot be used to definitively prove the course of an enzymatic mechanism, the collective results described here provide consistent evidence that iridoid synthase catalyzes cyclization of the iridoids via a Michael addition rather than a Diels-Alder reaction. Additionally, this work demonstrates that iridoid synthase can cyclize substrates other than 8-oxogeranial (**2**), suggesting the potential utility of this enzyme for enzymatic synthesis of new compounds. Understanding the mechanism of iridoid synthase cyclization now enables us to better predict which substrates this enzyme can cyclize. Further studies exploring the potential of this enzyme to synthesize new cyclic compounds from nonnatural substrates are currently under way.

## Significance

**Iridoid synthase is a recently discovered enzyme that catalyzes a noncanonical terpene cyclization reaction. The design and synthesis of two substrate analogs are used to probe the mechanism of iridoid synthase. Enzymatic assay of these substrate analogs, along with clues from the product profile of the native substrate, strongly suggest that iridoid synthase utilizes a Michael reaction to achieve cyclization, rather than a Diels-Alder reaction. Additionally, this work demonstrates that iridoid synthase can cyclize nonnative substrates, suggesting the potential utility of this enzyme for enzymatic synthesis of new compounds. This improved mechanistic understanding will facilitate the exploitation of the potential of iridoid synthase to synthesize new cyclic compounds from nonnatural substrates.**

## Experimental Procedures

All enzyme assays were carried out using 20 mM MOPS (pH 7.0) as buffer. The substrates were kept as 50 mM stocks in tetrahydrofuran (THF) at −20°C. Care was taken not to exceed THF concentrations higher than 0.5% in the presence of enzyme, as concentrations above 1% THF were found to affect activity adversely. The milligram-scale enzyme assays were carried out using an NADPH generation/regeneration system consisting of glucose-6-phosphate (G6P), glucose-6-phosphate dehydrogenase (G6PDH), and NADP^+^. Enzymatic rates for steady-state kinetic analysis of the iridoid synthase reactions were measured spectrophotometrically, monitoring NADPH consumption at 340 nm. For GC-MS analysis, reactions (200 μl) were set up in glass vials using 200 μM substrate, 600 μM NADPH, and 0.5 μg of purified protein and were terminated after 1 hr by adding 250 μl CH_2_Cl_2_. The organic phase was used directly for GC-MS analysis. Standard GC-MS spectra were recorded on an Agilent 6890N GC system equipped with a split/splitless injector and coupled to an Agilent 5973 MS detector. GC-MS-based accurate mass determination was performed on a Waters GCT system consisting of an Agilent 6890 Series GC system fitted with a split/splitless injector and coupled to a Waters GCT Classic Mass Spectrometer. For analysis by TLC, 150 μl of the organic phase was vacuum-concentrated to approximately 10 μl, spotted onto normal-phase TLC plates, run using 10:1 hexanes/ethyl acetate, and visualized with anisaldehyde stain. For kinetic studies, the absorbance at 340 nm of 200 μl assays was measured using a 96-well plate reader. Procedures for the synthesis of substrates **5** and **6**, along with all spectral characterization for synthetically and enzymatically generated products, are reported in the Supplemental Information.

## Author Contributions

S.L. carried out all syntheses, enzyme assays of **5** and **6**, and characterization of the enzymatic products. F.G.-F. cloned and expressed the enzyme version used in the assays, assayed substrate **2**, and performed the equilibrium experiments with open/closed forms of product **1**. S.B. provided intellectual support and supervision. N.H.S. conceived the design of substrates **5** and **6** as well as the initial synthetic strategy. S.E.O. was the overall supervisor. All authors contributed to the writing of the manuscript.

## Supplemental Information

Document S1. Supplemental Experimental Procedures and Figures S1–S3

Document S2. Article plus Supplemental Information
