# Supplementary material for: Conversion of Substrate Analogs Suggests a Michael Cyclization in Iridoid Biosynthesis
Source: Chem Biol. 2014 Nov 20;21(11):1452–6. doi: 10.1016/j.chembiol.2014.09.010 (PMC4245709; doi:10.1016/j.chembiol.2014.09.010)
Supplement: Document S1. Supplemental Experimental Procedures and Figures S1–S3 [file mmc1.pdf]

Chemistry & Biology, Volume *21*

Supplemental Information

Conversion of Substrate Analogs Suggests a Michael Cyclization in Iridoid Biosynthesis

Stephanie Lindner, Fernando Geu-Flores, Stefan Bräse, Nathaniel H. Sherden, and Sarah E. O'Connor

a

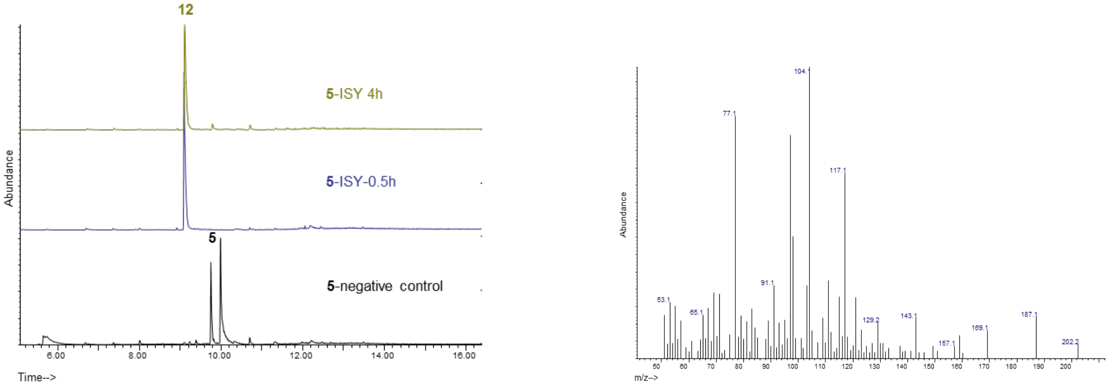
12
5-ISY 4h
5-ISY-0.5h
5
5-negative control
Abundance
Time-->
6.00
8.00
10.00
12.00
14.00
16.00
Abundance
m/z-->

b

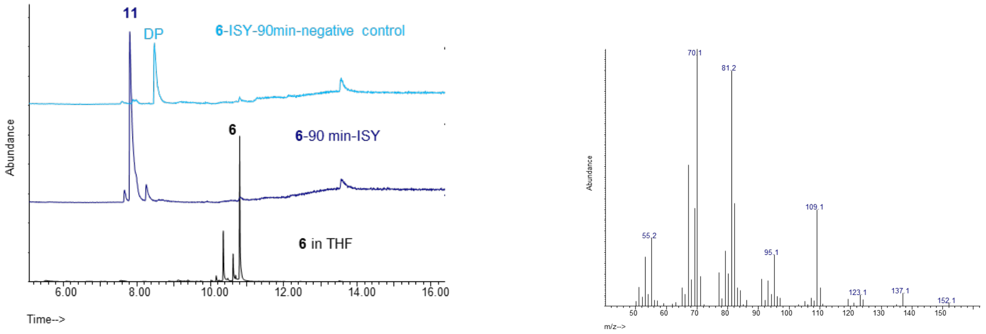
11
DP
6-ISY-90min-negative control
6-90 min-ISY
6
6 in THF
Abundance
Time-->
6.00
8.00
10.00
12.00
14.00
16.00
Abundance
m/z-->

c

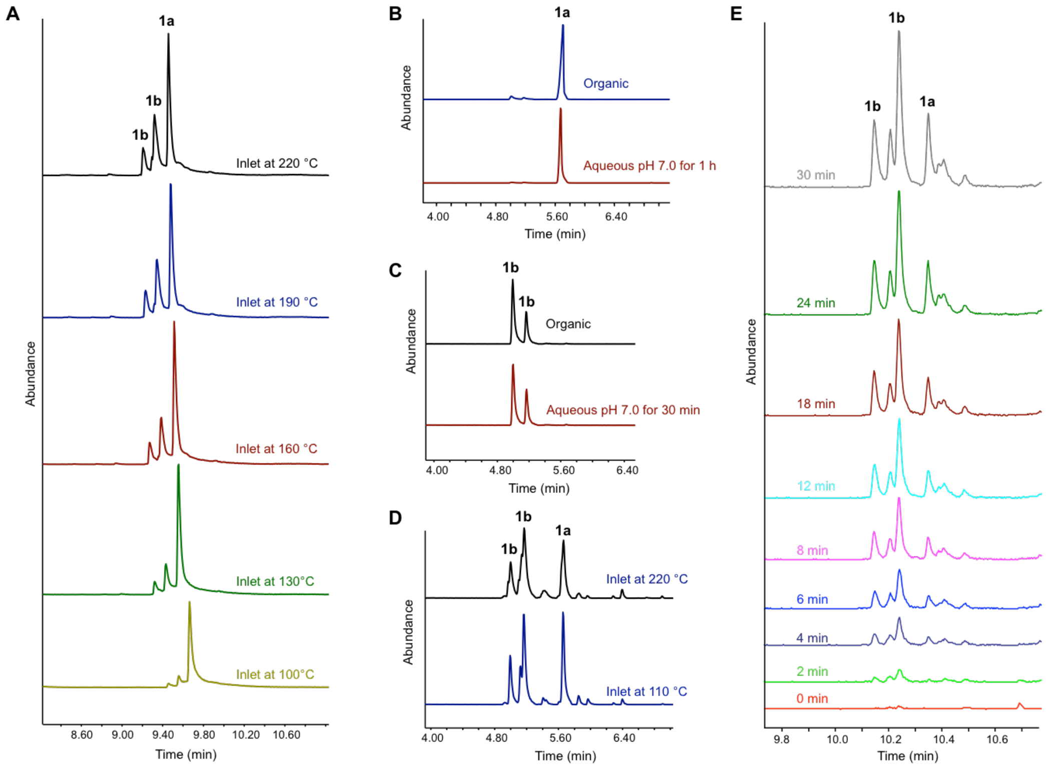
A
1a
1b
1b
Inlet at 220 °C
Inlet at 190 °C
Inlet at 160 °C
Inlet at 130°C
Inlet at 100°C
Abundance
8.60
9.00
9.40
9.80
10.20
10.60
Time (min)
B
1a
Organic
Aqueous pH 7.0 for 1 h
Abundance
4.00
4.80
5.60
6.40
Time (min)
C
1b
1b
Organic
Aqueous pH 7.0 for 30 min
Abundance
4.00
4.80
5.60
6.40
Time (min)
D
1b
1b
1a
Inlet at 220 °C
Inlet at 110 °C
Abundance
4.00
4.80
5.60
6.40
Time (min)
E
1b
1b
1a
30 min
24 min
18 min
12 min
8 min
6 min
4 min
2 min
0 min
Abundance
9.8
10.0
10.2
10.4
10.6
Time (min)

Supplementary Fig. 1, related to Figure 1. a. GC-MS chromatograms of iridoid synthase (ISY) with substrate **5**. The mass spectrum of the iridoid synthase product **12** is also shown. **b.** GC-MS chromatograms of iridoid synthase (ISY) with substrate **6**. The mass spectrum of the iridoid synthase product **11** is also shown. DP: Decomposition product. **c.** GC-MS studies on the open (1b) and closed (1b) forms of the iridoid synthase product **1.** For the analysis in panel A, a ZB-5 column was used; for the analysis in panels B-D, a DB-1 column was used; and for the analysis in panel E, an HP-5MS column was used (see details under General Experimental Procedures). (**A**) Pure closed synthetic nepetalactol (**1a**) in CH_2Cl_2 at various inlet temperatures. The amount of open form increases as inlet temperature increases, suggesting substantial thermal opening of the closed form in the inlet at temperatures above 100 °C. At inlet temperatures below 100 °C, separation was compromised (not shown). (**B**) Comparison of synthetic **1a** in CH_2Cl_2 ('organic') and **1a** incubated for 1 hour in neutral aqueous medium (20 mM MOPS, pH 7.0) and then extracted with CH_2Cl_2, using an inlet temperature of 110 °C. No extra peaks appear, indicating that closed form **1a** does not readily equilibrate to the open forms **1b** under the incubation conditions. (**C**) Analysis of the open forms **1b** in CH_2Cl_2 ('organic') or the open forms incubated in neutral aqueous medium (20 mM MOPS, pH 7.0) for 20 minutes and then extracted in CH_2Cl_2, using an inlet temperature of 110 °C. No extra peaks appear, indicating that open forms **1b** do not readily equilibrate to the close form **1a** under the incubation conditions. The open forms **1b** were purified as putative hydrates from the milligram-scale enzymatic assay described in reference 2 in the main text (fractions 5-12 from Column 2; see supplementary Figure 7 of the mentioned publication). (**D**) Product profiles of the ISY reaction at two different inlet temperatures. Both open and closed forms are still present when using the lower inlet temperature (110 °C). (**E**) Time course of the ISY reaction, analyzed using an inlet temperature of 110 °C. The product profile seems virtually identical at all times.

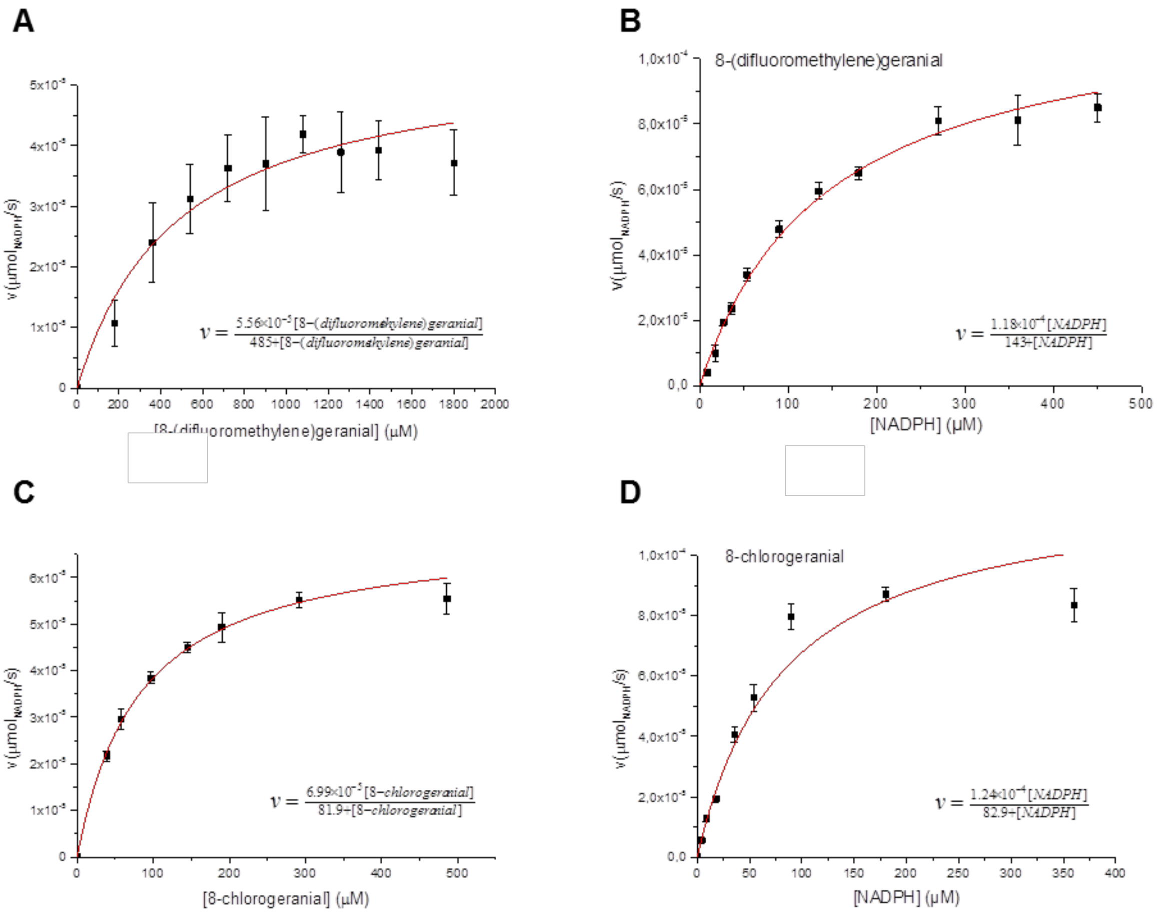

Supplementary Fig. S2, related to Figure 2. Steady-state kinetic analysis of the iridoid synthase reactions. A construct with a truncated N-terminus was used; this truncation has been used for all structural studies of the iridoid synthase homologue, progesterone beta reductase. Reaction rates were measured spectrophotometrically, monitoring NADPH consumption at 340 nm. Individual data points are averages of four replicates. Error bars represent standard errors (SE). The overlaid curves were obtained through non-linear regression (hyperbola), which yielded the rate equations inserted in each plot. (**A**) Saturation curve for 8-(difluoromethylene)geranial at a fixed NADPH concentration of 1 mM. $K_m = 485 \pm 160$ µM; $k_{cat} = 6.4 \pm 0.8$ s^{-1}. (**B**) Saturation curve for NADPH at a fixed 8-(difluoromethylene)geranial concentration of 2 mM. $K_m = 143 \pm 17$ µM; $k_{cat} = 13.6 \pm 1.2$ s^{-1}. (**C**) Saturation curve for 8-chlorogeranial at a fixed NADPH concentration of 160 µM. $K_m = 81.9 \pm 5.6$ µM; $k_{cat} = 8.1 \pm 0.5$ s^{-1}. (**D**) Saturation curve for NADPH at a fixed 8-chlorogeranial concentration of 300 µM. $K_m = 82.9 \pm 14.2$ µM; $k_{cat} = 8.1 \pm 0.5$ s^{-1}. For comparison, the kinetics of this truncated enzyme were measured with native substrate **2**. $K_M = 9.9 \pm 2.1$ µM, $k_{cat} = 1.4 \pm 0.1$ s^{-1}, $k_{cat}/K_M = 0.14$ s^{-1} μM^{-1} (The steady state kinetic parameters of native iridoid synthase with substrate **2** are: $K_M = 4.5 \pm 0.2$ μM, $k_{cat} = 1.6 \pm 0.1$ s^{-1}; for NADPH, $K_M = 4.7 \pm 0.7$ μM, $k_{cat} = 2.2 \pm 0.2$ s^{-1}).

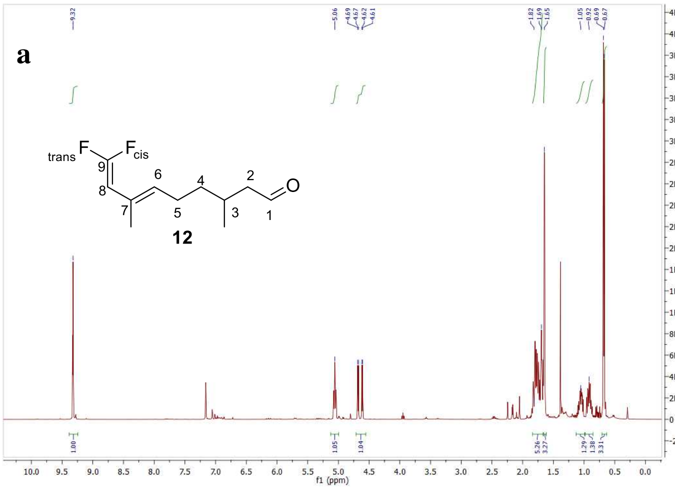
a
trans F
F cis
12
f1 (ppm)

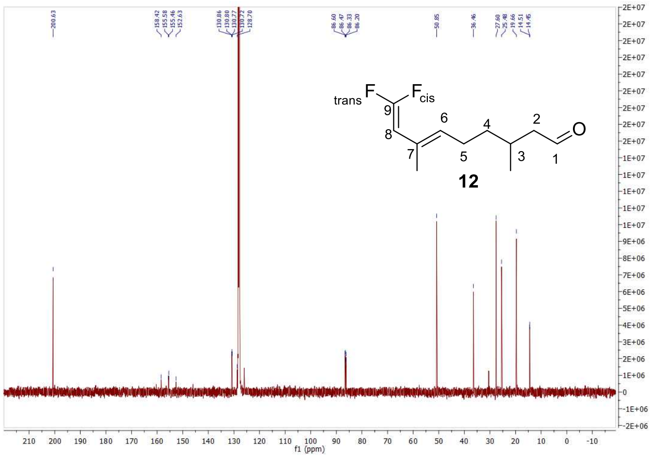
trans F
F cis
12
f1 (ppm)

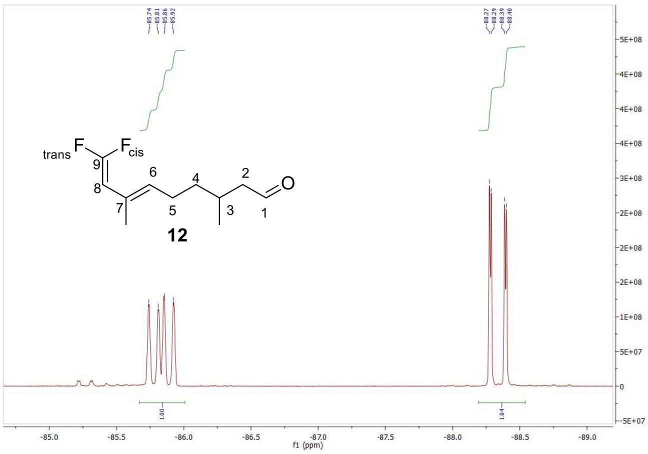
trans F
F cis
12
f1 (ppm)

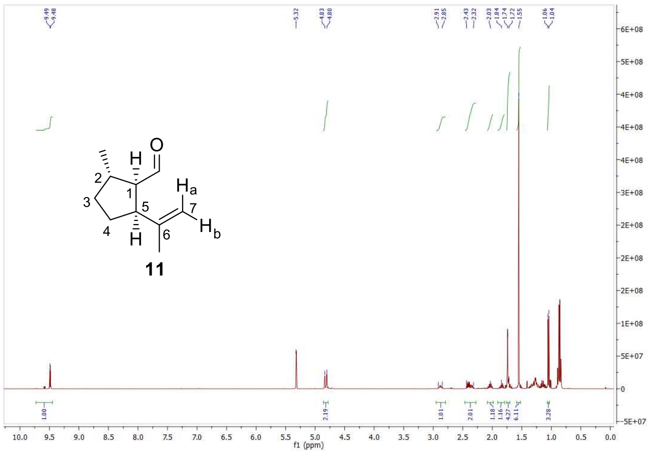
Ha
Hb
11
f1 (ppm)

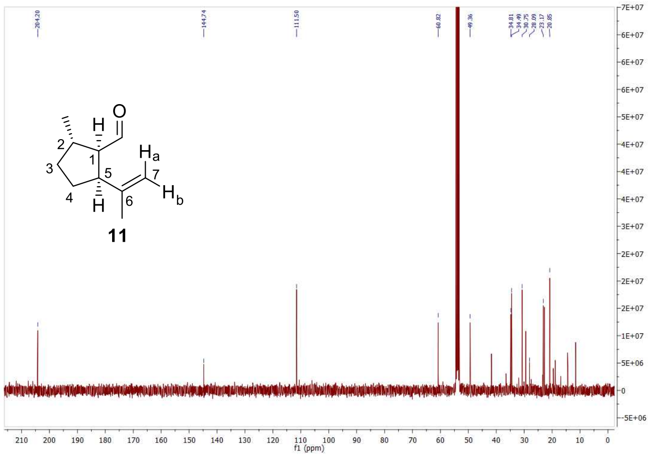
Ha
Hb
11
f1 (ppm)

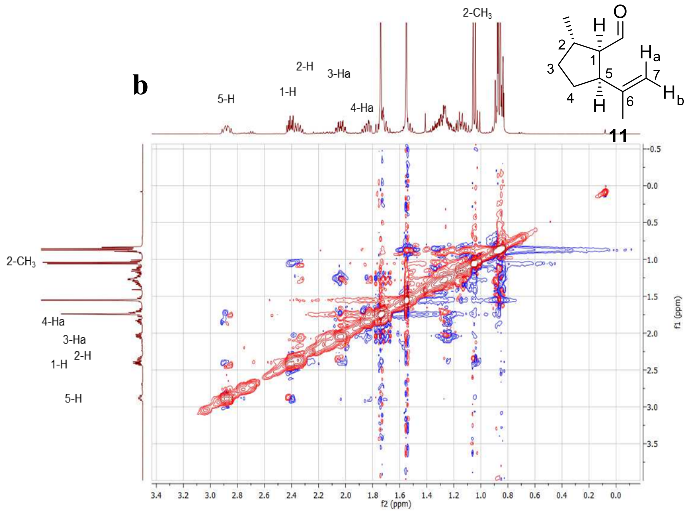
b
2-CH3
2-H
3-Ha
5-H
1-H
4-Ha
Ha
Hb
11
f2 (ppm)
f1 (ppm)

Supplementary Fig. S3, related to Figure 3. a. ^{1}H, ^{13}C and ^{19}F NMR spectra of the products resulting from incubation of substrate analogs with iridoid synthase. **b.** ^{1}H, ^{13}C and NOESY NMR spectra of the product resulting from incubation of **6** with iridoid synthase. NOE signals between 1-H and 5-H, and between 2-CH_3 and 1-H suggest the relative stereochemistry drawn.

Supplemental Experimental Procedures

A: General Experimental Procedures

Unless otherwise indicated, all reactions were monitored for completion by Thin Layer Chromatography (TLC). TLC was performed on Merck silica gel 60 F254 precoated (0.25 mm) glass backed plates, 20 x 20 cm, cut down to various sizes for use. TLC results were analyzed by UV fluorescence quenching on fluorescent (F254) plates, or with standard anisaldehyde, cerium sulfate or $KMnO_4$ stains. When necessary, reactions were heated in a silicone oil bath heated and regulated with an IKAmag hot-plate with a temperature-modulating thermocouple. Anhydrous DMF was obtained from Sigma-Aldrich in sure/seal™ bottles and used as received. The NMR solvents $CDCl_3$ and C_6D_6 were purchased from Sigma Aldrich and used as received. CD_2Cl_2 was purchased from Cambridge Isotope Laboratories, Inc. Petroleum ether was obtained from Fisher Scientific and is defined here as petroleum fractions that boil from 36–60 °C. All other standard solvents were purchased from Fisher Scientific and used as received. All filtrations were performed with either fritted glass Büchner funnels or cotton in traditional glass funnels. Activated charcoal (Sigma Aldrich) was used for decolorization. Celite® 545 (Sigma-Aldrich) was used for filtrations. Column chromatography was done using neutral silica gel (particle size D10: 33 µm, D50: 50 µm, D90: 70 µm [≈ 200 – 400 mesh]) from BDH Laboratory Supplies UK or on Florisil® (Sigma Aldrich). All other chemicals used for chemical synthesis were purchased from Sigma Aldrich and used as received.

^{1}H, ^{13}C and ^{19}F NMR spectra were recorded on a Bruker 400 MHz / 54 mm UltraShield Plus, long hold time automated NMR system. ^{1}H spectra are reported relative to residual $CHCl_3$ (at δ 7.26), CH_2Cl_2 (at δ 5.32) or C_6H_6 (at δ 7.16). The description of signals include: s = singlet, d = doublet, t = triplet, q = quartet, sx = sextet, m = multiplet, dd = doublet of doublets, ddd = doublet of dd, dt = doublet of triplets, dq = doublet of quartets. All couplings constants are absolute values.

^{13}C NMR spectra are reported relative to $CDCl_3$ (d = 77.16 ppm), CD_2Cl_2 (d = 53.84 ppm) and C_6D_6 (d = 128.06 ppm). The assignment of the atoms was made via partial characterization by 1D (^{1}H, ^{13}C, DEPT) and 2D (COSY, NOESY, HSQC) NMR. The signal structure was analyzed by DEPT and is described as follows: + = primary or tertiary C-atom (positive signal), – = secondary C-atom (negative signal), and $C_{quart.}$ = quaternary C-atom (no signal).

Standard GC-MS spectra were recorded on an Agilent 6890N GC system equipped with a split/splitless injector and coupled to an Agilent 5973 MS detector. Helium was used as carrier gas at 1 mL/min. The temperature of the inlet was 220 °C unless otherwise specified. For the analysis of the synthesized substrate analogs and their enzymatic products, separations were carried out with a Zebron ZB-5 HT column (30 m × 0.25 mm × 0.10 µm) using the following program: 5 min isothermal at 60 °C, then 20 °C/min gradient up to 150 °C, then 45°C/min gradient up to 280 °C (run time = 16.39 min). For the analysis of the open/closed forms of the native enzymatic product, separations were carried out either with the mentioned ZB-5 HT column and program or with an Agilent DB-1ht column (15 m × 0.25 mm × 0.10 µm) using the following program: 2 min isothermal at 60 °C, then 12 °C/min gradient up to 150 °C, then 45°C/min gradient up to 280 °C (run time = 14.39 min). For the reaction time course, separations were carried out using an Agilent HP-5MS column (30 m x 0.25 mm x 0.25 µm) and the same temperature program as the one used for the ZB-5 HT column (see above).

GC-MS based accurate mass determination was performed on a Waters GCT system consisting of an Agilent 6890 Series GC system fitted with split/splitless injector and coupled to a Waters GCT Classic Mass Spectrometer at Rothamsted

Research. In case where no accurate mass spectra could be measured due to the instability of the compound, the standard GC-MS is provided for characterization.

B: Synthesis of the test substrates

*Synthesis of 8-oxogeranyl acetal **16***

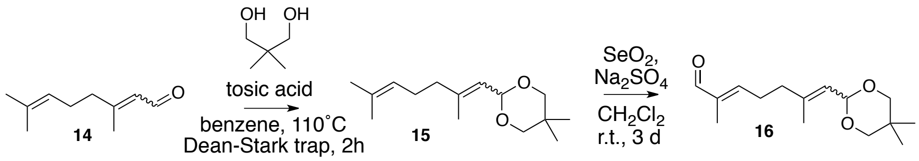

a) Synthesis of geranyl acetal [2-(2,6-dimethylhepta-1,5-dien-1-yl)-5,5-dimethyl-1,3-dioxane, **15**]:

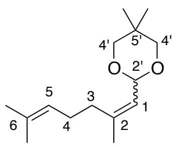

Tosic acid monohydrate (125 mg, 0.657 mmol, 0.01 equiv.) was added to a mixture of citral (10.0 g, 65.7 mmol, 1.00 equiv.) and 2,2-dimethylpropane-1,3-diol (20.5 g, 197 mmol, 3.00 equiv.) in benzene (100 mL). The flask was connected to a Dean-Stark trap and the mixture was refluxed at 110 °C for 2 h. After cooling to r.t. the reaction mixture was washed with 10% $NaHCO_{3(aq)}$ (3 × 150 mL) and the aqueous layers were back extracted with diethyl ether (100 mL). The combined organic phases were washed with brine (150 mL), dried over sodium sulfate, filtered and concentrated *in vacuo* affording crude **14** as a yellow oil in quantitative yield. The crude geranyl acetate product was used in subsequent reaction without farther purification. See I. Grosu, S. Mager, I. Batiu, *Rev. Roum. Chem.* **1995**, *40*, 1175-1182 for a prior synthesis.

^{1}H NMR (400 MHz, $CDCl_3$): δ = 5.34 (dd, ^3J = 6.3 Hz, ^4J = 1.1 Hz, 1 H, 1-H), 5.16–5.04 (m, 2 H, 5-H, 2'-H), 3.67–3.60 (m, 2 H, 4'-H_a), 3.54–3.46 (m, 2 H, 4'-H_b), 2.18–1.96 (m, 4 H, 3-H, 4-H), 1.76 (d, ^4J = 1.4 Hz, 3 H, 2_Z-CH_3), 1.74 (d, ^4J = 1.2 Hz, 3 H, 2_E-CH_3), 1.69 (s, 3 H, 6_{Za}-CH_3), 1.67 (s, 3 H, 6_{Ea}-CH_3), 1.61 (s, 3 H, 6_{Zb}-CH_3), 1.59 (s, 3 H, 6_{Eb}-CH_3), 1.22 (s, 3 H, 5'$_{Ea}$-CH_3), 1.21 (s, 3 H, 5'$_{Za}$-CH_3), 0.74 (s, 3 H, 5'$_{Eb}$-CH_3), 0.73 (s, 3 H, 5'$_{Zb}$-CH_3) ppm.

b) Synthesis of 8-oxogeranyl acetal [(2*E*,6*E*/*Z*)-7-(5,5-dimethyl-1,3-dioxan-2-yl)-2,6-dimethylhepta-2,6-dienal, **16**]:

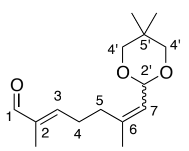

Crude geranyl acetal **15** (10.0 g, 42.0 mmol, 1.00 equiv.) was added to a mixture of SeO_2 (5.13 g, 46.2 mmol, 1.10 equiv.) and Na_2SO_4 (11.9 g, 84.0 mmol, 2.00 equiv.) in dichloromethane (100 mL) under nitrogen. The reaction mixture was stirred for three days at room temperature and then filtered through a thin pad of Celite® rinsing with dichloromethane. After concentrating the solution to roughly one-third of its original volume *in vacuo*, a 1:1 mixture of hexanes/ethyl acetate (200 mL) was added. The mixture was washed with 1:6:9 NaCl/bleach/H_2O (3 × 150 mL), saturated $NaHCO_{3(aq)}$ solution (3 × 150 mL), a 60 mg/mL solution of $Na_2SO_{3(aq)}$ (3 × 150 mL) and brine (2 × 150 mL). The organic phase was simultaneously dried and decolorized by stirring with a mixture of sodium sulfate and activated charcoal for one hour. The resulting black suspension was filtered through a thin pad of Celite® rinsing with ethyl acetate and concentrated *in vacuo*. Chromatography on silica (hexanes/EtOAc = 20/1 to 10/1) afforded 8-oxogeranyl acetal **16** as a separable mixture of two diastereomers (6*Z*:6*E* ≈ 1:1.5) in 11% yield (1.21 g, 4.77 mmol) as a yellow oil. The product could not be purified completely, and was partially still bound to selenium (seen by GC-MS).

6*Z*-isomer: ***R*$_f$** = 0.38 (hexanes/ethyl acetate = 5/1). **^{1}H NMR** (400 MHz, $CDCl_3$): δ = 9.39 (s, 1 H, 1-H), 6.54–6.48 (m, 1 H, 3-H), 5.42 (d, ^3J = 6.4 Hz, 1 H, 7-H), 5.03 (d, ^3J = 6.4 Hz, 1 H, 2'-H), 3.63 (dd, ^2J = 10.5 Hz, ^4J = 1.3 Hz, 2 H, 4'-H$_a$), 3.47 (d, ^2J = 10.5 Hz, 2 H, 4'-H$_b$), 2.52–2.26 (m, 4 H, 4-H, 5-H), 1.79 (d, ^4J = 1.5 Hz, 3 H, 6-CH_3), 1.76 (d, ^4J = 1.1 Hz, 3 H, 2-CH_3), 1.21 (s, 3 H, 5'-CH_{3a}), 0.73 (s, 3 H, 5'-CH_{3b}) ppm. **^{13}C NMR** (100 MHz, $CDCl_3$): δ = 195.4 (+, C-1), 153.6 (+, C-3), 141.6 (C_{quart}, C-2), 139.8 (C_{quart}, C-6), 124.5 (+, C-7), 98.5 (+, C-2'), 77.4 (–, C-4'$_a$), 77.2 (–, C-4'$_b$), 31.5 (–, C-5), 30.1 (C_{quart}, C-5'), 27.4 (–, C-4), 23.1 (+, 5'-CH_{3a}), 23.1 (+, 5'-CH_{3b}), 22.8 (+, 6-CH_3), 9.3 (+, 2-CH_3) ppm. The signal of C-4'$_b$ at 77.2 ppm is hidden by the solvent signal but could be determined via HSQC. **6*E*-isomer**: ***R*$_f$** = 0.33 (hexanes/ethyl acetate = 5/1). **^{1}H NMR** (400 MHz, $CDCl_3$): δ = 9.38 (s, 1 H, 1-H), 6.46 (tq, ^3J = 7.1 Hz, ^4J = 1.4 Hz, 1 H, 3-H), 5.37 (dq, ^3J = 6.3 Hz, ^4J = 1.3 Hz, 1 H, 7-H), 5.09 (d, ^3J = 6.3 Hz, 1 H, 2'-H), 3.67–3.44 (m, 4 H, 4'-H), 2.50 (q, ^3J = 7.4 Hz, 2 H, 4-H), 2.21 (t, ^3J = 7.8 Hz, 2 H, 5-H), 1.78 (d, ^4J = 1.1 Hz, 3 H, 6-CH_3), 1.74 (d, ^4J = 1.4 Hz, 3 H, 2-CH_3), 1.22 (s, 3 H, 5'-CH_{3a}), 0.74 (s, 3 H, 5'-CH_{3b}) ppm. **^{13}C NMR** (100 MHz, $CDCl_3$): δ = 195.3 (+, C-1), 153.6 (+, C-3), 141.3 (C_{quart}, C-2), 139.8 (C_{quart}, C-6), 123.1 (+, C-7), 98.8 (+, C-2'), 37.5 (–, C-5), 30.2 (C_{quart}, C-5'), 27.0 (–, C-4), 23.1 (+, 5'-CH_{3a}), 22.1 (+, 5'-CH_{3b}), 17.4 (+, 6-CH_3), 9.3 (+, 2-CH_3) ppm. The two signals of C-4' are hidden by the solvent signal and could not be determined.

Synthesis of the Diels-Alder test substrate 8-(difluoromethylene)geranial **5**

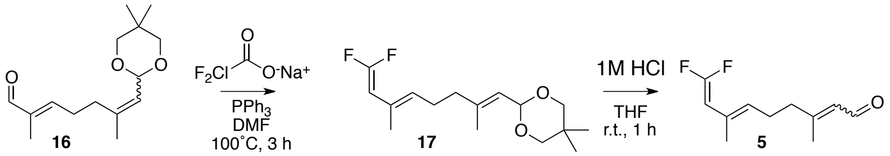

a) Synthesis of 8-(difluoromethylene)geranyl acetal [2-((1*E*/*Z*,5*E*)-8,8-difluoro-2,6-dimethylocta-1,5,7-trien-1-yl)-5,5-dimethyl-1,3-dioxane, **17**]:

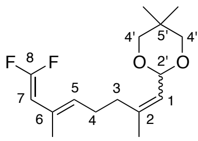

A solution of 8-oxogeranyl acetal **16** (mixture of both diastereomers, 400 mg, 1.59 mmol, 1.00 equiv.) in absolute DMF (1 mL) under nitrogen was added to a mixture of sodium chlorodifluoroacetate (725 mg, 4.76 mmol, 3.00 equiv.) and triphenylphosphine (1.25 g, 4.76 mmol, 3.00 equiv.) in absolute DMF (1 mL) under nitrogen at 30–40 °C. The reaction mixture was immediately heated up to 100 °C and stirred for 3 h at 100–105 °C, turning dark brown. After cooling to room temperature, diethyl ether (10 mL) and water (10 mL) were added. The resulting mixture was filtered, followed by rinsing with copious amounts of diethyl ether. The phases were separated and the aqueous phase was extracted with diethyl ether (3 × 10 mL). The combined organic phases were washed with brine (2 × 20 mL), dried over sodium sulfate, filtered and concentrated *in vacuo*. Chromatography on silica (hexanes/EtOAc = 50/1 to 25/1) afforded 8-(difluoromethylene)geranyl acetal **17** in 18% yield (79.8 mg, 0.279 mmol) as yellow oil. The reaction was also implemented using only the *6Z*-isomer or the *6E*-isomer of starting material **15**, respectively. Thus the diastereomers of **16** could be obtained separately.

1*Z*-isomer: ***R*$_f$** = 0.61 (hexanes/ethyl acetate = 5/1). **^{1}H NMR** (400 MHz, CD_2Cl_2): δ = 5.39 (t, ^3J = 7.0 Hz, 1 H, 5-H), 5.28–5.24 (m, 1 H, 1-H), 5.00 (d, ^3J = 6.4 Hz, 1 H, 2'-H), 4.89 (dd, $^3J_{HF}$ = 5.3, 26.9 Hz, 1 H, 7-H), 3.57 (d, ^2J = 10.5 Hz, 2 H, 4'-H$_a$), 3.45 (d, ^2J = 10.5 Hz, 2 H, 4'-H$_b$), 2.24–2.09 (m, 4 H, 3-H, 4-H), 1.83–1.81 (m, 3 H, 6-CH_3), 1.74 (d, 3 H, ^4J = 1.5 Hz, 2-CH_3), 1.16 (s, 3 H, 5'-CH_{3a}), 0.71 (s, 3 H, 5'-CH_{3b}) ppm. **^{13}C NMR** (100 MHz, CD_2Cl_2): δ = 142.0 (C_{quart}, C-2), 130.5 (C_{quart}, dd, $^3J_{CF}$ = 5.9, 8.8 Hz, C-6), 126.6 (m, C-2'), 124.1 (+, C-5), 98.9 (+, C-1), 86.3 (+, dd, $^2J_{CF}$ = 12.5, 27.1 Hz, C-7), 77.5 (–, 2 × C-4'), 32.7 (–, C-3), 30.1 (C_{quart}, C-5'), 26.8 (–, C-4), 23.2 (+, 5'-CH_{3a}), 23.1 (+, 5'-CH_{3b}), 22.0 (+, 2-CH_3), 14,6 (+, d,

$^4J_{CF}$ = 5.9 Hz, 6-CH_3) ppm. The signal of C-8 could not be detected, presumably because of low sample amount. **^{19}F NMR** (376 MHz, CD_2Cl_2): δ = –85.9 (ddd, $^2J_{FF}$ = 42.9 Hz, $^3J_{FH}$ = 26.6 Hz, $^5J_{FH}$ = 3.0 Hz, 1 F, F_{cis}), –85.24 (dd, $^2J_{FF}$ = 42.9 Hz, $^3J_{FH}$ = 3.2 Hz, 1 F, F_{trans}) ppm. **1*E*-isomer**: ***R*$_f$** = 0.61 (hexanes/ethyl acetate = 5/1). **^{1}H NMR** (400 MHz, CD_2Cl_2): δ = 5.39 (t, ^3J = 7.0 Hz, 1 H, 5-H), 5.27–5.23 (m, 1 H, 1-H), 5.02 (d, ^3J = 6.3 Hz, 1 H, 2'-H), 4.89 (dd, $^3J_{HF}$ = 5.4, 27.0 Hz, 1 H, 7-H), 3.57 (d, ^2J = 11.2 Hz, 2 H, 4'-H_a), 3.47 (d, ^2J = 11.0 Hz, 2 H, 4'-H_b), 2.25–2.02 (m, 4 H, 3-H, 4-H), 1.82–1.79 (m, 3 H, 6-CH_3), 1.71 (d, 3 H, ^4J = 1.4 Hz, 2-CH_3), 1.16 (s, 3 H, 5'-CH_{3a}), 0.71 (s, 3 H, 5'-CH_{3b}) ppm. **^{13}C NMR** (100 MHz, CD_2Cl_2): δ = 141.9 (C_{quart}, C-2), 130.5 (C_{quart}, dd, $^3J_{CF}$ = 5.9, 8.8 Hz, C-6), 126.6 (m, C-2'), 123.1 (+, C-5), 99.2 (+, C-1), 86.3 (+, dd, $^2J_{CF}$ = 12.5, 27.1 Hz, C-7), 77.5 (–, 2 × C-4'), 39.0 (–, C-3), 30.2 (C_{quart}, C-5'), 26.4 (–, C-4), 23.1 (+, 5'-CH_{3a}), 22.0 (+, 5'-CH_{3b}), 17.2 (+, 2-CH_3), 14.6 (+, d, $^4J_{CF}$ = 5.9 Hz, 6-CH_3) ppm. The signal of C-8 could not be detected. **^{19}F NMR** (376 MHz, CD_2Cl_2): δ = –85.8–(–86.1) (ddd, m, 1 F, F_{cis}), –88.9 (dd, $^2J_{FF}$ = 43.7 Hz, $^3J_{FH}$ = 5.5 Hz, 1 F, F_{trans}) ppm.

b) Synthesis of 8-(difluoromethylene)geranial [(6*E*)-9,9-difluoro-3,7-dimethylnona-2,6,8-trienal, **5**]:

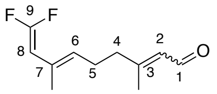

$HCl_{(aq)}$ (1 M, 1 mL) was added to a solution of 8-(difluoromethylene)geranyl acetal **17** (79.8 mg, 0.279 mmol, 1.00 equiv.) in tetrahydrofuran (2 mL). After stirring at room temperature for 1 h, a saturated solution of $NaHCO_{3(aq)}$ (2 mL) was carefully added and the mixture was extracted with ethyl acetate (3 × 5 mL). The merged organic phases were washed with $NaHCO_{3(aq)}$ (5 mL), brine (5 mL), dried over sodium sulfate, filtered and concentrated *in vacuo*. Chromatography on silica (hexanes/EtOAc = 10/1) afforded 8-(difluoromethylene)geranial **5** as an inseparable mixture of two diastereomers in 71% yield (39.6 mg, 0.198 mmol) as a yellow oil. The reaction was also implemented using only the 1*Z*-isomer or the 1*E*-isomer of starting material **17**; however, these reactions also formed a mixture of both diastereomers due to rapid isomerization. Compound **5** is quite reactive and is best stored in solution (e.g. tetrahydrofuran) at –20 °C.

***R*$_f$** = 0.46 (*Z*), 0.41 (*E*) (hexane/ethyl acetate = 5/1). **^{1}H NMR** (400 MHz, C_6D_6): both diastereomers: δ = 9.86 (d, ^3J = 7.8 Hz, 1 H, $CHO_{(E)}$), 9.82 (d, ^3J = 7.7 Hz, 1 H, $CHO_{(Z)}$), 5.79–5.73 (m, 2 H, 2-$H_{(E/Z)}$), 4.93–4.85 (m, 2 H, 6-$H_{(E/Z)}$), 4.61–4.50 (m, 2 H, 8-$H_{(E/Z)}$), 2.00 (t, ^3J = 7.5 Hz, 2 H, 4-$H_{(Z)}$), 1.81–1.58 (m, 6 H, 4-$H_{(E)}$, 4,5-$H_{(E/Z)}$), 1.57–1.54 (m, 3 H, 7-$CH_{3(E)}$), 1.53–1.50 (m, 3 H, 7-$CH_{3(Z)}$), 1.45 (s, 3 H, 3-$CH_{3(E)}$), 1.32 (s, 3 H, 3-$CH_{3(Z)}$) ppm. **^{13}C NMR** (100 MHz, C_6D_6): both diastereomers: δ = 189.6 (+, C-$1_{(E)}$), 189.1 (+, C-$1_{(Z)}$), 160.7 (C_{quart}, C-$3_{(Z)}$), 160.6 (C_{quart}, C-$3_{(E)}$), 155.6 (C_{quart}, dd, $^1J_{CF}$ = 297.8, 286.1 Hz, C-$9_{(Z)}$), 155.6 (C_{quart}, dd, $^1J_{CF}$ = 297.1, 285.4 Hz, C-$9_{(E)}$), 129.2–126.5 (C-$2_{(E/Z)}$, C-$6_{(E/Z)}$, C-$7_{(E/Z)}$), 86.1 (+, dd, $^3J_{CF}$ = 27.5, 12.0 Hz, C-$8_{(E)}$), 86.0 (+, dd, $^3J_{HF}$ = 27.5, 12.0 Hz, C-$8_{(Z)}$), 39.7 (–, C-$4_{(E)}$), 31.8 (–, C-$4_{(Z)}$), 26.8 (–, C-$5_{(Z)}$), 25.5 (–, C-$5_{(E)}$), 24.3 (+, 3-$CH_{3(Z)}$), 16.8 (+, 3-$CH_{3(E)}$), 14.4 (+, d, $^4J_{CF}$ = 5.8 Hz, 7-$CH_{3(E/Z)}$) ppm. The signals of C-2 and C-6 could only be detected *via* HSQC and not be unambiguously determined, because they are overlapped by the solvent signal (C_6D_6). C-7 could not be detected, but is also likely overlapped by the solvent signal. **^{19}F NMR** (376 MHz, C_6D_6): both diastereomers: δ = –84.95 (dd, $^2J_{FF}$ = 41.1 Hz, $^3J_{FH}$ = 26.2 Hz, 1 F, *cis*-$F_{(Z)}$), –85.24 (dd, $^2J_{FF}$ = 40.7 Hz, $^3J_{FH}$ = 27.0 Hz, 1 F, *cis*-$F_{(E)}$), –87.31–(–87.46) (m, 1 H, *trans*-$F_{(Z)}$), –87.61–(–87.77) (m, 1 H, *trans*-$F_{(E)}$) ppm. HR-GCMS calculated for $C_{11}H_{14}F_2O$ $[M]^+$: 200.1013, found 200.1019 (*Z*), 200.1015 (*E*).

*Synthesis of the Michael addition test substrate 8-chlorogeranial **6***

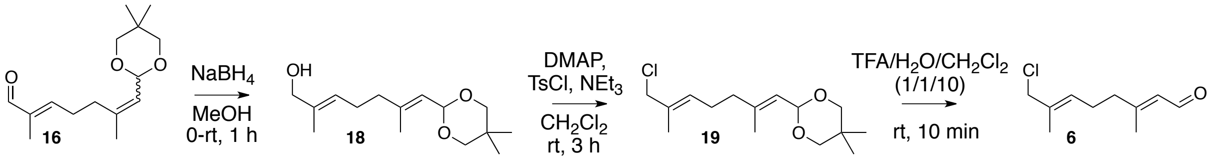

a) Synthesis of 8-hydroxygeranyl acetal [(2*E*,6*E*/*Z*)-7-(5,5-dimethyl-1,3-dioxan-2-yl)-2,6-dimethylhepta-2,6-dien-1-ol, **18**]:

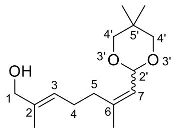

Sodium borohydride (122 mg, 3.23 mmol, 2.00 equiv.) was added to a solution of 8-oxogeranyl acetal **16** (408 mmol, 1.61 mmol, 1.00 equiv.) in methanol (8 mL) at 0 °C, turning the mixture light yellow, then red. The reaction mixture was allowed to warm up to room temperature where stirring was continued for 1.5 h. Additional sodium borohydride (122 mg, 3.23 mmol, 2.00 equiv.) was added and the mixture was stirred for another 10 minutes. Saturated $NaHCO_{3(aq)}$ (8 mL) was added slowly and the reaction mixture was extracted with dichloromethane (3 × 10 mL). The combined organic layers were washed with brine (2 × 10 mL), dried over sodium sulfate, filtered and concentrated *in vacuo*. Chromatography on silica (hexanes/EtOAc = 5/1) afforded 8-hydroxygeranyl acetal **18** as a mixture of two diastereomers in 36% yield (146 mg, 0.574 mmol) as yellow oil. The reaction was also implemented using only the 6*Z*-isomer or the 6*E*-isomer of starting material **16**, respectively. Thus the diastereomers of **18** could be obtained separately. It should be noted, that the starting material bound to selenium didn't react.

***6Z*-isomer**: $\boldsymbol{R}_\mathbf{f}$ = 0.40 (hexanes/ethyl acetate = 2/1). **^{1}H NMR** (400 MHz, CD_2Cl_2): δ = 5.39–5.33 (m, 1 H, 3-H), 5.28–5.24 (m, 1 H, 7-H), 5.00 (d, ^3J = 6.9 Hz, 1 H, 2'-H), 3.92 (d, ^3J = 5.3 Hz, 2 H, 1-H), 3.58 (dd, ^2J = 9.9 Hz, ^4J = 1.3 Hz, 2 H, 4'-H_a), 3.45 (dd, ^2J = 11.2 Hz, ^4J = 0.8 Hz, 2 H, 4'-H_b), 2.18–2.14 (m, 4 H, 4-H, 5-H), 1.75 (d, ^4J = 1.5 Hz, 3 H, 6-CH_3), 1.63 (d, ^4J = 1.3 Hz, 3 H, 2-CH_3), 1.16 (s, 3 H, 5'-CH_{3a}), 0.71 (s, 3 H, 5'-CH_{3b}) ppm. **^{13}C NMR** (100 MHz, CD_2Cl_2): δ = 141.8 (C_{quart}, C-6), 136.4 (C_{quart}, C-2), 125.1 (+, C-5), 123.9 (+, C-7), 99.0 (+, C-2'), 77.4 (–, 2 × C-4'), 69.0 (–, C-1), 32.7 (–, C-5), 30.1 (C_{quart}, C-5'), 26.0 (–, C-4), 23.2 (+, 5'-CH_{3a}), 23.0 (+, 5'-CH_{3b}), 22.0 (+, 6-CH_3), 13.8 (+, 2-CH_3) ppm. ***6E*-isomer**: $\boldsymbol{R}_\mathbf{f}$ = 0.36 (hexanes/ethyl acetate = 2/1). **^{1}H NMR** (400 MHz, CD_2Cl_2): δ = 5.35 (sxd, ^3J = 7.0 Hz, ^4J = 1.3 Hz, 1 H, 3-H), 5.23 (sxd, ^3J = 6.4 Hz, ^4J = 1.3 Hz, 1 H, 7-H), 5.04 (d, ^3J = 6.4 Hz, 1 H, 2'-H), 3.94 (s, 2 H, 1-H), 3.58 (dd, ^2J = 9.9 Hz, ^4J = 1.3 Hz, 2 H, 4'-H_a), 3.47 (dd, ^2J = 11.2 Hz, ^4J = 0.8 Hz, 2 H, 4'-H_b), 2.21–2.03 (m, 4 H, 4-H, 5-H), 1.71 (d, ^4J = 1.3 Hz, 3 H, 6-CH_3), 1.64 (s, 3 H, 2-CH_3), 1.17 (s, 3 H, 5'-CH_{3a}), 0.71 (s, 3 H, 5'-CH_{3b}) ppm. **^{13}C NMR** (100 MHz, CD_2Cl_2): δ = 141.9 (C_{quart}, C-6), 136.0 (C_{quart}, C-2), 125.4 (+, C-5), 123.2 (+, C-7), 99.2 (+, C-2'), 77.5 (–, 2 × C-4'), 69.1 (–, C-1), 39.1 (–, C-5), 30.2 (C_{quart}, C-5'), 25.9 (–, C-4), 23.1 (+, 5'-CH_{3a}), 22.0 (+, 5'-CH_{3b}), 17.1 (+, 6-CH_3), 14.2 (+, 2-CH_3) ppm.

b) Synthesis of 8-chlorogeranyl acetal [2-((1*E*,5*E*)-7-chloro-2,6-dimethylhepta-1,5-dien-1-yl)-5,5-dimethyl-1,3-dioxane, **19**]:

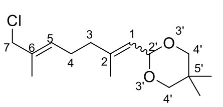

DMAP (43.9 mg, 0.359 mmol, 0.625 equiv.), tosyl chloride (131 mg, 0.689 mmol, 1.20 equiv.) and NEt_3 (80 µL, 58.1 mg, 0.574 mmol, 1.00 equiv.) were sequentially added to a solution of 8-hydroxygeranyl acetal **18** (146 mg, 0.574 mmol, 1.00 equiv.) in dichloromethane (3 mL) under nitrogen. After stirring for 3 h at room temperature, the solvent was removed *in vacuo*. Chromatography on Florisil® (hexanes/EtOAc = 10/1 + 5% NEt_3) afforded 8-chlorogeranyl acetal **19** as an inseparable mixture of two diastereomers in 45% yield (69.7 mg, 0.255 mmol) as yellow oil. The reaction was also implemented using only the 6*E*-isomer of starting material **18**. Thus the 1*E*-diastereomer of **19** could be obtained analytically pure.

$\boldsymbol{R}_\mathbf{f}$ = 0.64 (hexanes/ethyl acetate = 10/1). **1*E*-isomer**: **^{1}H NMR** (400 MHz, CD_2Cl_2): δ = 5.54 (dt, ^3J = 7.0 Hz, ^4J = 1.1 Hz, 1 H, 5-H), 5.25 (sxd, ^3J = 6.3 Hz, ^4J = 1.3 Hz 1 H, 1-H), 5.03 (d, ^3J = 6.3 Hz, 1 H, 2'-H), 4.02 (s, 2 H, 7-H), 3.57 (d,

^2J = 11.2 Hz, 2 H, 4'-H$_a$), 3.47 (d, ^2J = 10.9 Hz, 4'-H$_b$), 2.21–2.02 (m, 4 H, 3-H, 4-H), 1.72 (d, ^4J = 1.1 Hz, 3 H, 6-CH$_3$), 1.70 (d, ^4J = 1.3 Hz, 3 H, 2-CH$_3$), 1.15 (s, 3 H, 5'-CH$_{3a}$), 0.71 (s, 3 H, 5'-CH$_{3b}$) ppm. **^{13}C NMR** (100 MHz, CD_2Cl_2): δ = 141.83 (C$_{quart}$, C-2), 132.6 (C$_{quart}$, C-6), 130.4 (+, C-5), 123.1 (+, C-1), 99.2 (+, C-2'), 77.5 (–, 2 × C-4'), 52.9 (–, C-7), 38.7 (–, C-3), 30.2 (C$_{quart}$, C-5'), 26.6 (–, C-4), 23.1 (+, 5'-CH$_{3a}$), 22.0 (+, 5'-CH$_{3b}$), 17.2 (+, 2-CH$_3$), 14.2 (+, 6-CH$_3$) ppm.

c) Synthesis of 8-chlorogeranial [(6*E*,2*E*/*Z*)-8-chloro-3,7-dimethylocta-2,6-dienal, **6**]: A mixture of TFA/H_2O (1:1, 0.4 mL)

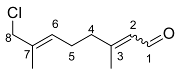

was added to a solution of 8-chlorogeranyl acetal **19** (69.7 mg, 0.255 mmol, 1.00 equiv.) in dichloromethane (2 mL). After stirring for 10 minutes, saturated $NaHCO_{3(aq)}$ (2 mL) was added to the reaction carefully. The mixture was immediately extracted with dichloromethane (3 × 5 mL), the combined organic layers were dried over sodium sulfate, filtered and concentrated *in vacuo*. Chromatography on Florisil® (hexanes/EtOAc = 10/1 + 5% NEt_3) afforded 8-chlorogeranial **6** as an inseparable mixture of two diastereomers in 69% yield (32.7 mg, 0.175 mmol) as yellow oil. The reaction was also implemented using only the 1*E*-isomer of starting material **6**, also leading to a mixture of both diastereomers due to rapid isomerization. The molecule reacted easily, resulting in the rearranged product with a mass of 168.1, which was obtained as an inseparable byproduct.

***R*f** = 0.28 (hexanes/ethyl acetate = 10/1). **Major isomer (2*E*): ^{1}H NMR** (400 MHz, CD_2Cl_2): δ = 9.98 (d, ^3J = 8.0 Hz, 1 H, 1-H), 5.83 (dd, ^3J = 8.0 Hz, ^4J = 1.2 Hz, 1 H, 2-H), 5.58–5.48 (m, 1 H, 6-H), 4.02 (s, 2 H, 8-H), 2.29–2.25 (m, 4 H, 4-H, 5-H), 2.16 (d, ^4J = 1.2 Hz, 3 H, 3-CH$_3$), 1.75–1.73 (m, 3 H, 7-CH$_3$) ppm. **^{13}C NMR** (100 MHz, CD_2Cl_2): δ = 191.3 (+, C-1), 163.2 (C$_{quart}$, C-3), 133.5 (C$_{quart}$, C-7), 129.2 (+, C-6), 127.8 (+, C-2), 52.5 (–, C-8), 40.0 (–, C-4), 26.0 (–, C-5), 17.7 (+, 3-CH$_3$), 14.3 (+, 7-CH$_3$) ppm. HR-GCMS calculated for $C_9H_{12}O$ $[M-CH_3]^+$: 171.0577, found 171.0585 (*Z*), 171.0577 (*E*).

C: Enzymatic assays

All enzyme assays were carried out using 20 mM MOPS pH 7.0 as buffer. The substrates were kept as 50 mM stocks in tetrahydrofuran (THF) at −20 °C. During the preparation of master mixes, care was taken not to exceed THF concentrations higher than 0.5% in the presence of enzyme, as concentrations above 1% THF were found to affect activity adversely. The batch of THF used contained a small amount of inhibitor of peroxide formation (butylated hydroxytoluene, BHT, added by the solvent manufacturer), which is visible as a small peak at approximately 10.35 min in the GC chromatograms of extracted enzymatic products.

Cloning and expression of truncated iridoid synthase

The USER-compatible vector pET28au was created by amplifying the GFP coding sequence from pEAQ-*HT*-GFP [Sainsbury et al. (2011) *Plant Biotechnol J* **7**:682] using primers **AAAAAAGGATCCGCTGAGGCCTTAATTAAATGACTAGCAAAGGAGAAGAACTTTTCAC** and **AAAAAAAAGCTTGCTGAGGTTTAATTAATTATTTGTATAGTTGATCCATGCC** and cloning the PCR product into pET28a via the BamHI and HindIII restriction sites. The coding sequence of iridoid synthase lacking 30 amino acids N-terminally was amplified from pDEST17-*ISY* [see reference 2 from main text] using primers GGCCTTAAUTGAAAACCTGTATTTTCAGGGCGTGGCACTAGTAGTAGGAGTCACC **and** GGTTTAAUCTAAGGAATAAACCTATAATCCCTCATCTTATCAAT and USER-cloned into Nt.BbvCI/PacI-digested pET28au [Nour-Eldin et al. (2010) *Methods Mol Biol* **35**:e55]. The tagged, truncated protein was expressed from the resulting plasmid and purified by

affinity purification as described in reference 2 of the main text. Apart from six consecutive His residues, the N-terminal protein tag (MGSSHHHHHHSSGLVPRGSHMASMTGGQQMGRGSAEALIENLYFQG) also provided a TEV cleavage site (NLYFQG), but all attempts to remove the tag using TEV protease were unsuccessful.

Amino acid sequence of iridoid synthase. Residues crossed out were deleted in the truncated construct.:
~~mswwwkrsigagknlpnqnkengvcksyks~~valvvgvtgivgsslaevlklpdtpggpwkvygvarrpcpvwlakkpveyiqc
dvsdnqetisklsplkdithifyvswigsedcqtnatmfknilnsvipnasnlqhvclqtgikhyfgifeegskvvphdspft
edlprlnvpnfyhdledilyeetgknnltwsvhrpalvfgfspcsmmnivstlcvyatickhenkalvypgsknswncyadav
dadlvaeheiwaavdpkaknqvlncnngdvfkwkhiwkklaeefgiemvgyvegkeqvslaelmkdkdqvwdeivkknnlvpt
klkeiaafwfadiafcsenlissmnkskelgflgfrnsmksfvscidkmrdyrfip

TLC- and GC–MS-based assays (small-scale)

Reactions (200 µL) were set up in glass vials using 200 µM substrate, 600 µM NADPH, 20 mM MOPS pH 7.0, and 0.5 µg of purified protein, and were terminated after 1 h by adding 250 µL CH_2Cl_2. The organic phase was used directly for GC–MS analysis. For analysis by TLC, 150 µL of the organic phase was vacuum-concentrated to approximately 10 µL, spotted onto normal-phase TLC plates, run using 10:1 hexanes: ethyl acetate, and visualized with anisaldehyde stain. For the reaction time course, an analogous 1mL reaction containing 500 µM 8-oxogeranial, 1.5 mM NADPH, 20 mM MOPS pH 7.0, and 180 ng of purified protein was set up. The reaction was terminated at different times by adding 100 µL of ethyl acetate to 50-µL aliquots, and the organic phase was used directly for GC–MS analysis.

Milligram-scale (large-scale) assays

The milligram-scale enzyme assays were carried out using an NADPH-generation/regeneration system consisting of glucose-6-phosphate (G6P), glucose-6-phosphate dehydrogenase (G6PDH) and $NADP^+$.

a) Enzymatic synthesis of (E)-9,9-difluoro-3,7-dimethylnona-6,8-dienal **12**

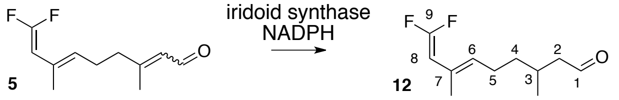

The reaction of 8-(difluoromethylene)geranial **5** with the iridoid synthase was set up in 375 mL of 50 mM MOPS pH 7.0 using 937 µg of purified enzyme, 15 mg of 8-(difluoromethylene)geranial **5** (0.0750 mmol), 110 mg of G6P, 250 U of G6PDH and 17.0 mg of $NADP^+$. The reaction was monitored continuously by TLC using a short-wave ultraviolet lamp for detection, and went to completion after 1 h. The products were extracted with dichloromethane (3 × 200 mL), 1 h later. The combined organic layers were washed with H_2O (200 mL), dried over sodium sulfate, filtered and concentrated *in vacuo* afforded compound **12** as light yellow oil (14.9 mg, 0.0737 mmol, 98%).

^{1}H NMR (400 MHz, C_6D_6): δ = 9.32 (t, ^3J = 1.9 Hz, 1 H, 1-H), 5.64 (dd, $^3J_{HF}$ = 5.1, 26.9 Hz, 1 H, 8-H), 5.06 (t, ^3J = 7.2 Hz, 1 H, 6-H), 1.84–1.66 (m, 5 H, 2-H, 3-H, 5-H), 1.66–1.62 (m, 3 H, 7-CH_3), 1.10–0.83 (m, 2 H, 4-H), 0.68 (d, ^3J = 6.4 Hz, 3 H, 3-CH_3) ppm. **^{13}C NMR** (100 MHz, C_6D_6): δ = 200.6 (+, C-1), 155.5 (C_{quart}, dd, $^1J_{CF}$ = 285.2, 297.2 Hz, C-9), 130.8 (+, dd, $^4J_{CF}$ = 8.8, 5.4 Hz, C-6), 128.7 (C_{quart}, C-7), 86.4 (+, dd, $^3J_{CF}$ = 27.2, 12.5 Hz, C-8), 50.8 (–, C-2), 36.5 (–, C-4), 27.6 (+, C-3) 25.5 (–, C-5), 19.7 (+, 3-CH_3), 14.5 (+, d, $^4J_{CF}$ = 5.9 Hz, 7-CH_3) ppm. **^{19}F NMR** (376 MHz, C_6D_6): δ = –85.83 (dd,

$^2J_{FF}$ = 42.7 Hz, $^3J_{FH}$ = 26.7 Hz, 1 F, *cis*-F), –85.24 (dd, $^2J_{FF}$ = 42.7 Hz, $^3J_{FH}$ = 5.00 Hz, 1 F, *trans*-F) ppm. See selected spectra in Supplementary Figure 3. HR-GCMS calculated for $C_{11}H_{16}F_2O$ $[M]^+$: 202.1169, found 202.1163.

b) Enzymatic synthesis of (1R,2S,5S)-2-methyl-5-(prop-1-en-2-yl)cyclopentanecarbaldehyde **11**

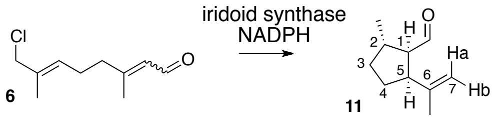

The reaction of 8-chlorogeranial **6** with the iridoid synthase was set up in 290 mL of 50 mM MOPS pH 7.0 using 722 µg of purified enzyme, 10.8 mg of 8-chlorogeranial **6** (0.0581 mmol), 85.5 mg of G6P, 250 U of G6PDH and 13.1 mg of $NADP^+$. The reaction was monitored continuously by TLC using a short-wave ultraviolet lamp for detection, and went to completion after 1 h. The products were extracted with dichloromethane (3 × 200 mL), 1 h later. The combined organic layers were washed with H_2O (200 mL), dried over sodium sulfate, filtered and concentrated under reduced pressure Due to the fact, that the product is probably volatile, the pressure was kept over 600 mbar. The crude product (11.5 mg) was purified *via* chromatography on silica (petrol ether/Et_2O = 20/1) affording compound **11** as colorless oil in about 57% yield (5 mg, 0.0329 mmol).
^{1}H NMR (400 MHz, CD_2Cl_2): δ = 9.48 (d, ^3J = 3.8 Hz, 1 H, CHO), 4.85–4.82 (m, 1 H, 7-H_a), 4.81–4.79 (m, 1 H, 7-H_b), 2.92–2.82 (m, 1 H, 5-H), 2.42–2.38 (m, 1 H, 1-H), 2.37–2.30 (m, 1 H, 2-H), 2.08–1.99 (m, 1 H, 3-H_a), 1.88–1.80 (m, 1 H, 4-H_a), 1.75–1.73 (m, 3 H, 6-CH_3), 1.73–1.71 (m, 1 H, 4-H_b), 1.28–1.26 (m, 1 H, 3-H_b), 1.04 (d, ^3J = 6.7 Hz, 3 H, 2-CH_3) ppm. **^{13}C NMR** (100 MHz, CD_2Cl_2): δ = 204.2 (+, CHO), 144.7 (C_{quart}, C-6), 111.5 (–, C-7), 60.8 (+, C-1), 49.3 (+, C-5), 34.8 (+, C-2), 34.5 (–, C-3), 30.7 (–, C-4), 23.2 (+, 6-CH_3), 20.8 (+, 2-CH_3) ppm. The signal for 3-H_b was overlapped by solvent signals. Since the product is volatile, the solvent could not be totally evaporated. However, HSQC and COSY showed coupling at this position. NOESY showed coupling between 1-H and 5-H as well as between 1-H and 2-CH_3. See selected spectra in Supplementary Figure 4. HR-GCMS calculated for $C_{10}H_{16}O$ $[M]^+$: 152.1201, found 152.1197.

It should be mentioned that the molecule **6** decomposes in aqueous solution within approximately 3 hours. However, time courses showed that the enzymatic reaction is complete within several minutes. Since the enzymatic reaction is so much faster than the decomposition, mechanistic studies were not affected.

Spectrophotometry-based assays (kinetic and substrate specificity studies)

For kinetic studies, the absorbance at 340 nm of 200 µL assays was measured using a 96-well plate reader. See the legend to Supplementary Fig.5 for further experimental details. Data were collected for 3–15 min, with individual measurements being taken for a single sample every 10 s when using full plate. The measured absorbances were plotted manually on Excel and the initial delta absorption/delta time values were calculated for the linear part of each reaction. NADPH consumption rates were calculated from these delta absorption/delta time values considering background NADPH decay and an extinction-coefficient-like value (dependent on assay volume) calculated separately. The Michaelis-Menten curve was fitted using Origin software.
