# Supplementary material for: Conversion of Substrate Analogs Suggests a Michael Cyclization in Iridoid Biosynthesis
Source: Chem Biol. 2014 Nov 20;21(11):1452–6. doi: 10.1016/j.chembiol.2014.09.010 (PMC4245709; doi:10.1016/j.chembiol.2014.09.010)
Supplement: Document S2. Article plus Supplemental Information [file mmc2.pdf]

Chemistry & Biology
Brief Communication

Conversion of Substrate Analogs Suggests a Michael Cyclization in Iridoid Biosynthesis

Stephanie Lindner,[1,2] Fernando Geu-Flores,[2] Stefan Bräse,[2,3] Nathaniel H. Sherden,[1,*] and Sarah E. O'Connor[1,*]
[1]Department of Biological Chemistry, The John Innes Centre, Norwich NR4 7UH, UK
[2]Institute of Organic Chemistry, Karlsruhe Institute of Technology, Fritz-Haber-Weg 6, 76131 Karlsruhe, Germany
[3]Institute of Toxicology and Genetics, Hermann-von-Helmholtz-Platz 1, 76344 Eggenstein-Leopoldshafen, Germany
*Correspondence: nat.sherden@jic.ac.uk (N.H.S.), sarah.oconnor@jic.ac.uk (S.E.O.)
http://dx.doi.org/10.1016/j.chembiol.2014.09.010
This is an open access article under the CC BY license (http://creativecommons.org/licenses/by/3.0/).

SUMMARY

The core structure of the iridoid monoterpenes is formed by a unique cyclization reaction. The enzyme that catalyzes this reaction, iridoid synthase, is mechanistically distinct from other terpene cyclases. Here we describe the synthesis of two substrate analogs to probe the mechanism of iridoid synthase. Enzymatic assay of these substrate analogs along with clues from the product profile of the native substrate strongly suggest that iridoid synthase utilizes a Michael reaction to achieve cyclization. This improved mechanistic understanding will facilitate the exploitation of the potential of iridoid synthase to synthesize new cyclic compounds from nonnatural substrates.

INTRODUCTION

The iridoids are a distinct class of approximately 600 monoterpenes that display a broad range of pharmacological and agrochemical activities (Tundis et al., 2008; Dewhirst et al., 2010; Dinda et al., 2011). We recently reported the discovery of iridoid synthase, the enzyme that produces nepetalactol (**1a**), the common biosynthetic precursor for all iridoids (Geu-Flores et al., 2012). Notably, this enzyme is mechanistically distinct from canonical terpene synthases (Uesato et al., 1983; Uesato et al., 1984; Uesato et al., 1987). Instead of forming a reactive cationic species from geranyl pyrophosphate (Figure 1A) (Degenhardt et al., 2009; Chen et al., 2011; Kim et al., 2012), iridoid synthase catalyzes cyclization that is triggered by reduction of 8-oxogeranial (**2**) to form enol or enolate intermediate **3**. Intermediate **3** is poised to cyclize to form nepetalactol (**1a**) by either an inverse electron demand hetero Diels-Alder (for examples of enzymatic Diels-Alder reactions, see Kim et al., 2012) or a Michael reaction (for examples of enzymatic Michael reactions, see Kusebauch et al., 2009; Bretschneider et al., 2013) (Figure 1B). Here we describe the synthesis and enzymatic assay of two substrate analogs designed to probe which reaction pathway iridoid synthase favors for cyclization. On the basis of these studies, along with clues from the product profile of the native substrate, it appears that iridoid synthase utilizes a Michael addition reaction mechanism for cyclization of the iridoid class of natural products. This provides an essential piece of the mechanistic puzzle of how the iridoid scaffold is constructed.

RESULTS AND DISCUSSION

After iridoid synthase reduces 8-oxogeranial (**2**) using nicotinamide adenine dinucleotide phosphate (NADPH) as hydride (H^-) donor, enol or enolate intermediate **3** is formed (Figure 1B). The existence of reaction intermediate **3** is supported by the identification of reduced aldehyde **4**, the more stable tautomer of **3**, as a minor product in the iridoid synthase catalyzed reaction (Geu-Flores et al., 2012). Moreover, the formation of an enol intermediate is entirely consistent with the proposed mechanism of progesterone-β-reductase (Thorn et al., 2008; Bauer et al., 2010), which displays high sequence similarity to iridoid synthase (67% amino acid identity compared with *Digitalis purpurea* P5bR2). Once formed, **3** can cyclize to form the characteristic bicyclic 5-6 ring iridoid framework of nepetalactol (**1a**). However, the specific mechanism of this cyclization is cryptic. In one scenario, cyclization could occur by a stepwise Michael reaction, forming the 5-membered ring first, with subsequent cyclization to the lactol (Figure 1B, blue arrows). Alternatively, the reaction could proceed via an inverse electron demand hetero Diels-Alder reaction (Figure 1B, red arrows).

To distinguish between these two mechanistic possibilities, two substrate analogs theoretically capable of cyclization by iridoid synthase were synthesized. One substrate, compound **5**, was designed to disfavor the Michael mechanism while favoring a Diels-Alder reaction; the other, compound **6**, strongly disfavored the Diels-Alder reaction while favoring the Michael reaction (Figure 2). Provided that both can be accommodated within the enzyme active site, cyclization of only one of these substrates by the enzyme would suggest the more likely reaction mechanism for the native substrate.

Iridoid synthase is predicted to reduce compound **5** to enol/enolate intermediate **7**. Intermediate **7** harbors a diene with electron withdrawing groups (fluorine) and a dienophile with an electron donating group (OH or O^-) and could therefore undergo an inverse electron demand Diels-Alder to form product **8** (Figure 2A); precedent for fluorinated dienes in enhancing Diels-Alder reactions exists (Kaz'mina et al., 1984; Roversi et al., 2002; Vogel et al., 2007). In contrast, the Michael addition with substrate **5** entails formation of a carbanion species (Figure 2A), which is far less stable than the enol or enolate species that would occur with the native substrate (Figure 1B). Although the

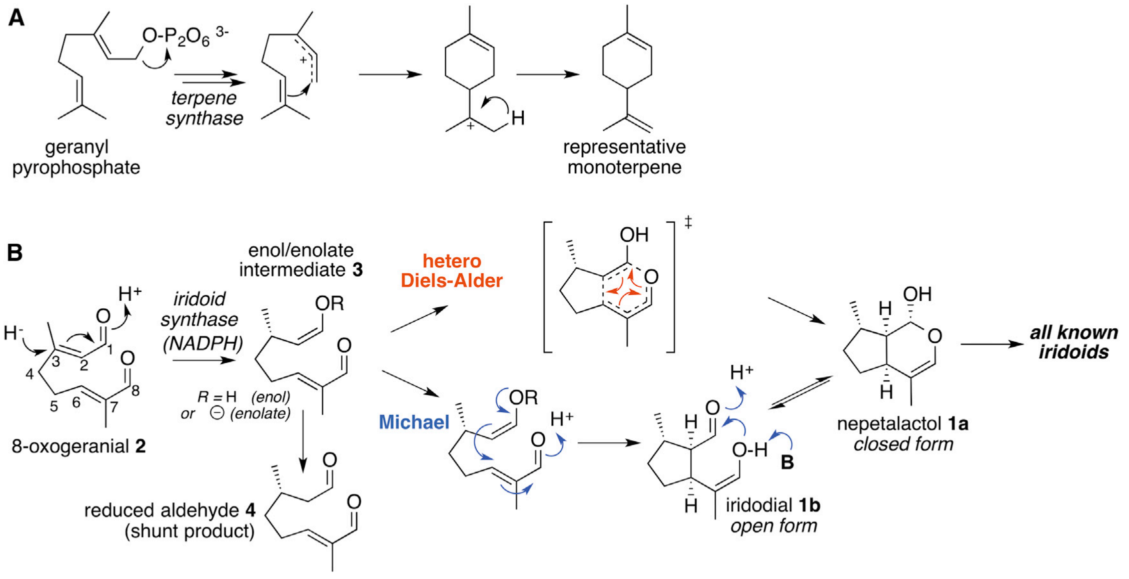

Figure 1. Terpene Cyclization

(A) Representative canonical terpene cyclization mechanism.

(B) Iridoid synthase uses 8-oxogeranial (**2**) as a substrate. A hydride from NADPH reduces the substrate to an enol or enolate **3**, which can then cyclize to **1a**. Two possible cyclization mechanisms are possible for iridoid synthase: a concerted hetero Diels-Alder (red arrows) and a stepwise Michael addition (blue arrows).

carbanion could be somewhat stabilized because of the inductive electron-withdrawing effects of the fluorine atoms, this effect is greatly mitigated because of electron-pair repulsions between the carbanion and the fluorine lone pairs (Zhang et al., 2012). Altogether, compared with the native substrate **2**, **5** is a much weaker candidate for a Michael addition. Thus the formation of cyclization product **8** upon incubation of iridoid synthase with substrate **5** would suggest that the enzyme utilizes a pericyclic reaction mechanism.

Candidate **6** is intended to undergo conjugate reduction in the enzyme to form intermediate **10**, which is primed to perform an S_N2' conjugate addition to give cyclization product **11** (Figure 2B). It is highly unlikely for intermediate **10** to undergo a Diels-Alder equivalent reaction (a halo-Alder-ene); we have found no precedent for such a reaction. Therefore, formation of cyclized product **11** would suggest that the enzyme utilizes a Michael reaction mechanism.

The synthesis of both **5** and **6** started with the acetalization of citral (**14**), followed by the allylic oxidation of one methyl group using stoichiometric amounts of SeO_2 to yield **16** (Figure 3; Supplemental Information available online). Ensuing difluoromethylenation with sodium chlorodifluoroacetate followed by hydrolysis of the acetal led to the Diels-Alder test substrate, 8-(difluoromethylene)geranial (**5**). The Michael test substrate, 8-chlorogeranial (**6**), was obtained from **16** by reduction of the aldehyde using sodium borohydride, chlorination with tosyl chloride followed by deacetalization (Figure 3; Supplemental Information). Compounds **5** and **6** were incubated with iridoid synthase and product formation was assessed by gas chromatography-mass spectrometry (GC-MS). The major products for both enzymatic reactions were also isolated, purified, and then characterized by nuclear magnetic resonance (NMR), further validating the structures of the enzymatic products (SI).

Upon incubation of iridoid synthase with substrate **5**, the linear reduction product **12** was observed (Figure 2A, red compound). This indicates that iridoid synthase is catalytically competent with **5**, despite the perturbations to the native substrate structure. Additionally, the lack of observable cyclized product **8** shows that the enzyme does not favor the Diels-Alder cyclization mechanism for which this substrate was designed. In contrast, when iridoid synthase was incubated with compound **6**, cyclized product **11** could be cleanly isolated (Figure 2B, blue compound). Given that a pericyclic reaction for compound **6** is highly disfavored, it seems most likely that the observed cyclization occurs via the Michael reaction. Nuclear Overhauser effect spectroscopy NMR spectra suggest that the product has the relative stereochemistry shown (Supplemental Information), which matches that of the native enzyme product **1b**.

Compounds **5** and **6** were subjected to steady-state kinetic analysis (Supplemental Information). Compound **5** (K_M = 485 ± 160 μM, k_{cat} = 6.4 ± 0.8 s^{-1}, k_{cat}/K_M = 0.013 s μM^{-1}; Supplemental Information) had a catalytic efficiency 8-fold less than that observed for compound **6** (K_M = 81.9 ± 5.6 μM, k_{cat} = 8.1 ± 0.5 s^{-1}, k_{cat}/K_M = 0.099 s μM^{-1}; Supplemental Information). Although both **5** and **6** had lower catalytic efficiencies than that observed for natural substrate **2** (K_M = 9.9 ± 2.1 μM, k_{cat} = 1.4 ± 0.1 s^{-1}, k_{cat}/K_M = 0.14 s^{-1} μM^{-1}), the steady-state kinetic measurements confirm that both **5** and **6** are competent substrates, though only substrate **6** was cyclized. For these studies, an enzyme with a truncation at the N terminus was used, which increases the structural stability of the nearest iridoid synthase homolog, progesterone beta-reductase. This truncation has recently been shown to affect kinetic parameters for progesterone beta-reductase (Rudolph et al., 2014). Therefore, kinetic parameters for **2** were remeasured using this truncated enzyme.

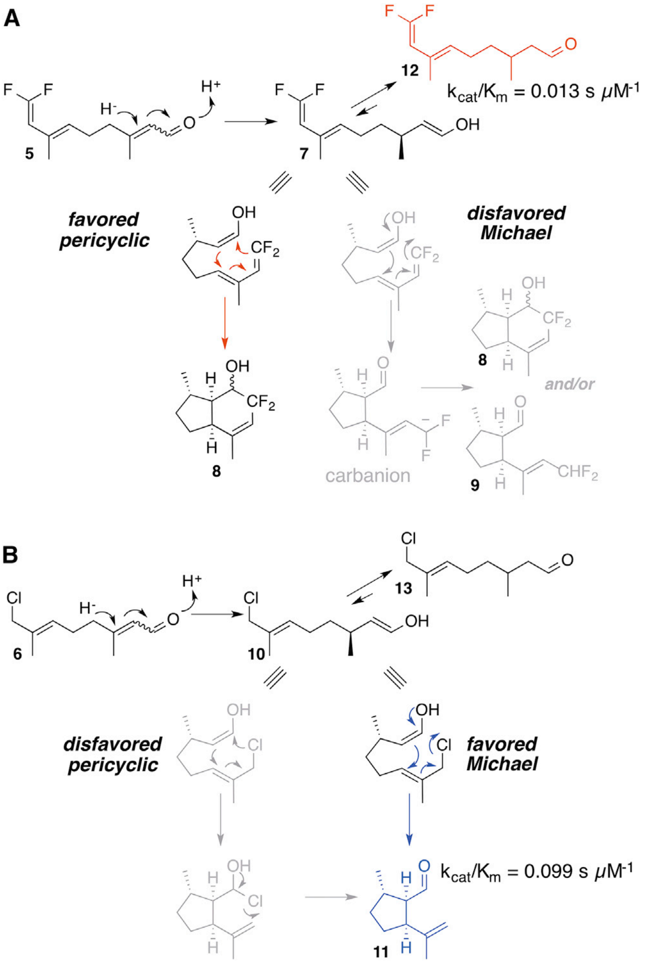

Figure 2. Substrate Analogs

(A) Compound **5** is poised to undergo a Diels-Alder reaction (red arrows) upon enol (or enolate) formation, while the Michael reaction is disfavored.

(B) Compound **6** is predisposed to undergo a Michael reaction (blue arrows) and is unlikely to undergo the Diels-Alder related Halo-Alder-ene pericyclic reaction. Disfavored mechanisms are shown in gray. Compounds **7** and **10** are shown in enol forms. Enzymatic products that were isolated are shown in red, along with corresponding k_{cat} and K_M values.

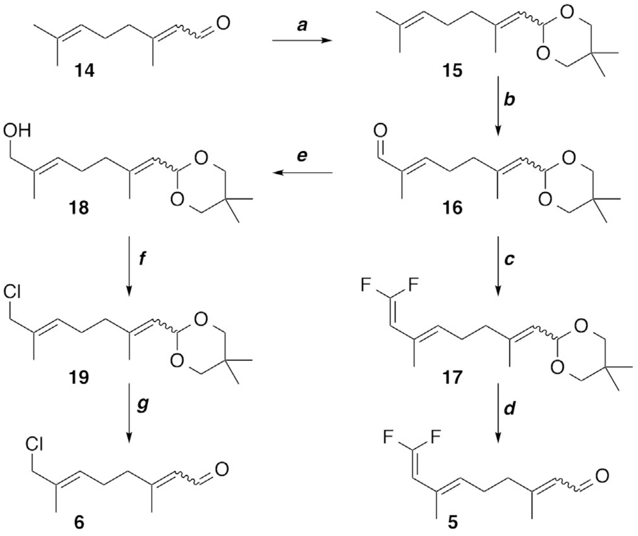

Figure 3. Synthetic Procedures for Compounds 5 and 6

(A) *p*-Toluenesulfonic acid monohydrate, 2,2-dimethylpropane-1,3-diol, benzene, 110°C, 2 hr (quant.)

(B) SeO_2, Na_2SO_4, dichloromethane, reaction time (r.t.), 2 d (11%).

(C) Sodium chlorodifluoroacetate, PPh_3, dimethylformamide, 100°C, 3 hr (18%).

(D) HCl, THF, r.t., 1 hr (71%).

(E) $NaBH_4$, MeOH, 0°C-r.t., 1.5 hr (36%).

(F) Tosyl chloride, 4-dimethylaminopyridine, NEt_3, dichloromethane, r.t., 3 hr (45%).

(G) Trifluoroacetic acid/H_2O (1:1), dichloromethane, r.t., 10 min (69%).

Full details are provided in the Supplemental Information.

For comparison, kinetic parameters for the full-length enzyme with **2** are K_M = 4.5 ± 0.2 μM, k_{cat} = 1.6 ± 0.1 s^{-1}, k_{cat}/K_M = 0.36 s^{-1} μM^{-1}.

The mechanistic implications drawn from substrate analogs must be interpreted with caution. For example, both analogs **5** and **6** had a higher K_M than native substrate **2**, but the difference was more marked for **5**, which might be indicative of an impaired binding to the active site. Additionally, the electronic properties of both analogs could be modulated by hydrogen bonding interactions with the enzyme, thereby altering the propensity of the compounds to cyclize via a Diels-Alder or Michael reaction. Ideally, comparison of nonenzymatic cyclization reactions with enzyme-catalyzed reactions would provide more insight into the baseline reactivity of these compounds. Unfortunately, we were unable to chemically cyclize these compounds. After chemical reduction of a more stable and synthetically accessible analog of **5** (9,9-difluoro-2,6-dimethylnona-2,6,8-trienal) using L-selectride to generate the reactive enol/enolate, we only obtained the alcohol (9,9-difluoro-2,6-dimethylnona-2,6,8-trien-1-ol). Reduction using Stryker's reagent in combination with LiCl or TMSCl led to an unidentifiable product mixture. Efforts to generate a protected enol species that could be subjected to chemical conditions favorable for a Diels-Alder reaction were unsuccessful. Efforts to assess whether **6** could cyclize nonenzymatically were complicated by the propensity of **6** to rearrange in solution. Despite these caveats, the results from enzymatic assay with the two substrate analogs are consistent: substrate **5**, which is primed for a Diels-Alder reaction, failed to cyclize, whereas substrate **6**, primed for a Michael reaction, did cyclize. Therefore, it seems reasonable to conclude that the cyclization step of iridoid synthase likely operates via a Michael addition reaction mechanism.

Finally, it is prudent to consider whether the product distribution that results from native substrate **2** also supports this mechanism. The Michael reaction proceeds via the open form of nepetalactol (**1a**), iridodial (**1b**), while the Diels-Alder proceeds directly to the closed form **1a** (Figure 1B). The native iridoid synthase cyclization product appears as a mixture of the closed and open forms, **1a** and **1b**, as evidenced by GC-MS and TLC (Geu-Flores et al., 2012). We reported in our initial experiments that **1a** and **1b** are in equilibrium (Geu-Flores et al., 2012), which would mean that the presence of both the open and closed forms provides no insight into a mechanistic hypothesis. We have now performed a more detailed analysis of the

product distribution of **1a** and **1b,** which demonstrates that the amount of open form observed is in fact greatly dependent upon the temperature of the GC inlet (low GC inlet temperatures have shown the stability of the open form), as well as on its usage history (Dawson et al., 1989). With this knowledge at hand, it is clear that the open and closed forms of **1** equilibrate on a much slower timescale than previously assumed (see the Supplemental Information for detailed information and data). Therefore, we can now conclude that both the open and closed forms are produced in the enzymatic reaction. Because the Diels-Alder mechanism does not involve the open form **1b**, we would be less likely to observe the open form if the enzyme used a pericyclic cyclization. The presence of both open and closed forms of **1** is also more consistent with a cyclization mechanism utilizing the Michael reaction. Although the slow equilibrium would suggest that the stereochemistry at the hemi-acetal carbon of the closed form **1a** could provide mechanistic insight into the nature of the cyclization reaction, epimerization can also occur via acid-catalyzed loss of lactolic OH to give an oxocarbenium intermediate. Therefore, we have not considered the stereochemistry of **1a** at this carbon as supportive of one mechanism over the other.

In synthetic systems, intramolecular cyclization of dicarbonyl substrates to form the iridoid scaffold has utilized both Diels-Alder and Michael addition mechanisms. For example, a domino Knoevenagel-hetero-Diels-Alder reaction has been employed to form the iridoid scaffold (Tietze and Bartels, 1991), and an enol ether derivative of a trialdehyde substrate also cyclized immediately via an intramolecular inverse electron demand Diels-Alder to yield an iridoid derivative (Tietze et al., 1980; Tietze et al., 1982; Tietze, 1983). However, intramolecular cyclization of dicarbonyl substrates to yield iridoids has also been achieved via Michael reaction using a Jørgensen-Hayashi catalyst (Marqués-López et al., 2009), and a reductive Michael cyclization of a keto aldehyde has been reported (Yang et al., 2005). Although the inherent chemical reactivity of the linear iridoid precursor is compatible with both reactions, our studies suggest that nature utilizes the Michael reaction.

Iridoid synthase joins a growing list of diverse enzymes that catalyze unusual terpene cyclization reactions (Itoh et al., 2010; Shoyama et al., 2012; Xu et al., 2012). Although substrate probes and product identities cannot be used to definitively prove the course of an enzymatic mechanism, the collective results described here provide consistent evidence that iridoid synthase catalyzes cyclization of the iridoids via a Michael addition rather than a Diels-Alder reaction. Additionally, this work demonstrates that iridoid synthase can cyclize substrates other than 8-oxogeranial (**2**), suggesting the potential utility of this enzyme for enzymatic synthesis of new compounds. Understanding the mechanism of iridoid synthase cyclization now enables us to better predict which substrates this enzyme can cyclize. Further studies exploring the potential of this enzyme to synthesize new cyclic compounds from nonnatural substrates are currently under way.

SIGNIFICANCE

Iridoid synthase is a recently discovered enzyme that catalyzes a noncanonical terpene cyclization reaction. The design and synthesis of two substrate analogs are used to probe the mechanism of iridoid synthase. Enzymatic assay of these substrate analogs, along with clues from the product profile of the native substrate, strongly suggest that iridoid synthase utilizes a Michael reaction to achieve cyclization, rather than a Diels-Alder reaction. Additionally, this work demonstrates that iridoid synthase can cyclize nonnative substrates, suggesting the potential utility of this enzyme for enzymatic synthesis of new compounds. This improved mechanistic understanding will facilitate the exploitation of the potential of iridoid synthase to synthesize new cyclic compounds from nonnatural substrates.

EXPERIMENTAL PROCEDURES

All enzyme assays were carried out using 20 mM MOPS (pH 7.0) as buffer. The substrates were kept as 50 mM stocks in tetrahydrofuran (THF) at -20°C. Care was taken not to exceed THF concentrations higher than 0.5% in the presence of enzyme, as concentrations above 1% THF were found to affect activity adversely. The milligram-scale enzyme assays were carried out using an NADPH generation/regeneration system consisting of glucose-6-phosphate (G6P), glucose-6-phosphate dehydrogenase (G6PDH), and $NADP^+$. Enzymatic rates for steady-state kinetic analysis of the iridoid synthase reactions were measured spectrophotometrically, monitoring NADPH consumption at 340 nm. For GC-MS analysis, reactions (200 μl) were set up in glass vials using 200 μM substrate, 600 μM NADPH, and 0.5 μg of purified protein and were terminated after 1 hr by adding 250 μl CH_2Cl_2. The organic phase was used directly for GC-MS analysis. Standard GC-MS spectra were recorded on an Agilent 6890N GC system equipped with a split/splitless injector and coupled to an Agilent 5973 MS detector. GC-MS-based accurate mass determination was performed on a Waters GCT system consisting of an Agilent 6890 Series GC system fitted with a split/splitless injector and coupled to a Waters GCT Classic Mass Spectrometer. For analysis by TLC, 150 μl of the organic phase was vacuum-concentrated to approximately 10 μl, spotted onto normal-phase TLC plates, run using 10:1 hexanes/ethyl acetate, and visualized with anisaldehyde stain. For kinetic studies, the absorbance at 340 nm of 200 μl assays was measured using a 96-well plate reader. Procedures for the synthesis of substrates **5** and **6**, along with all spectral characterization for synthetically and enzymatically generated products, are reported in the Supplemental Information.

SUPPLEMENTAL INFORMATION

Supplemental Information includes Supplemental Experimental Procedures and three figures and can be found with this article online at http://dx.doi.org/10.1016/j.chembiol.2014.09.010.

AUTHOR CONTRIBUTIONS

S.L. carried out all syntheses, enzyme assays of **5** and **6**, and characterization of the enzymatic products. F.G.-F. cloned and expressed the enzyme version used in the assays, assayed substrate **2**, and performed the equilibrium experiments with open/closed forms of product **1**. S.B. provided intellectual support and supervision. N.H.S. conceived the design of substrates **5** and **6** as well as the initial synthetic strategy. S.E.O. was the overall supervisor. All authors contributed to the writing of the manuscript.

ACKNOWLEDGMENTS

We gratefully acknowledge the Biotechnology and Biological Sciences Research Council (BB/J009091/1), Landesgraduiertenförderung Baden-Württemberg, and the German Academic Exchange Service for funding. We thank John Pickett and his research team, especially Keith Chamberlain, for helpful discussions regarding the equilibrium of **1** between open and closed forms and for bringing our attention to Dawson et al. (1989). We thank Sarah Hayes for initial work on the synthesis of **5** and **6**.

Received: May 8, 2014
Revised: August 29, 2014
Accepted: September 2, 2014
Published: October 23, 2014

REFERENCES

Bauer, P., Munkert, J., Brydziun, M., Burda, E., Müller-Uri, F., Gröger, H., Muller, Y.A., and Kreis, W. (2010). Highly conserved progesterone 5 β-reductase genes (P5 β R) from 5 β-cardenolide-free and 5 β-cardenolide-producing angiosperms. Phytochemistry *71*, 1495–1505.

Bretschneider, T., Heim, J.B., Heine, D., Winkler, R., Busch, B., Kusebauch, B., Stehle, T., Zocher, G., and Hertweck, C. (2013). Vinylogous chain branching catalysed by a dedicated polyketide synthase module. Nature *502*, 124–128.

Chen, F., Tholl, D., Bohlmann, J., and Pichersky, E. (2011). The family of terpene synthases in plants: a mid-size family of genes for specialized metabolism that is highly diversified throughout the kingdom. Plant J. *66*, 212–229.

Dawson, G.W., Janes, N.F., Mudd, A., Pickett, J.A., Slawin, A.M.Z., Wadhams, L.J., and Williams, D.J. (1989). The aphid sex pheromone. Pure Appl. Chem. *61*, 555–558.

Degenhardt, J., Köllner, T.G., and Gershenzon, J. (2009). Monoterpene and sesquiterpene synthases and the origin of terpene skeletal diversity in plants. Phytochemistry *70*, 1621–1637.

Dewhirst, S.Y., Pickett, J.A., and Hardie, J. (2010). Aphid pheromones. In Vitamins and Hormones, *Volume 83*, L. Gerald, ed. (San Diego: Academic Press), pp. 551–574.

Dinda, B., Debnath, S., and Banik, R. (2011). Naturally occurring iridoids and secoiridoids. An updated review, part 4. Chem. Pharm. Bull. (Tokyo) *59*, 803–833.

Geu-Flores, F., Sherden, N.H., Courdavault, V., Burlat, V., Glenn, W.S., Wu, C., Nims, E., Cui, Y., and O'Connor, S.E. (2012). An alternative route to cyclic terpenes by reductive cyclization in iridoid biosynthesis. Nature *492*, 138–142.

Itoh, T., Tokunaga, K., Matsuda, Y., Fujii, I., Abe, I., Ebizuka, Y., and Kushiro, T. (2010). Reconstitution of a fungal meroterpenoid biosynthesis reveals the involvement of a novel family of terpene cyclases. Nat. Chem. *2*, 858–864.

Kaz'mina, N.B., Mysov, E.I., Kvasov, B.A., Antipin, M.Y., and Struchkov, Y.T. (1984). Reaction of hexafluorobutadiene with α-methoxystyrene. Bull. Acad. Sci. USSR *3*, 2511–2518.

Kim, H.J., Ruszczycky, M.W., and Liu, H.-W. (2012). Current developments and challenges in the search for a naturally selected Diels-Alderase. Curr. Opin. Chem. Biol. *16*, 124–131.

Kusebauch, B., Busch, B., Scherlach, K., Roth, M., and Hertweck, C. (2009). Polyketide-chain branching by an enzymatic Michael addition. Angew. Chem. Int. Ed. Engl. *48*, 5001–5004.

Marqués-López, E., Herrera, R.P., Marks, T., Jacobs, W.C., Könning, D., de Figueiredo, R.M., and Christmann, M. (2009). Crossed intramolecular Rauhut-Currier-type reactions via dienamine activation. Org. Lett. *11*, 4116–4119.

Roversi, E., Scopelliti, R., Solari, E., Estoppey, R., Vogel, P., Braña, P., Menéndez, B., and Sordo, J.A. (2002). The hetero-Diels-Alder addition of sulfur dioxide to 1-fluorobuta-1,3-dienes: the sofa conformations preferred by 6-fluorosultines (6-fluoro-3,6-dihydro-1,2-oxathiin-2-oxides) enjoy enthalpic and conformational Anomeric effects. Chemistry *8*, 1336–1355.

Rudolph, K., Bauer, P., Schmid, B., Mueller-Uri, F., and Kreis, W. (2014). Truncation of N-terminal regions of Digitalis lanata progesterone 5β-reductase alters catalytic efficiency and substrate preference. Biochimie *101*, 31–38.

Shoyama, Y., Tamada, T., Kurihara, K., Takeuchi, A., Taura, F., Arai, S., Blaber, M., Shoyama, Y., Morimoto, S., and Kuroki, R. (2012). Structure and function of Δ1-tetrahydrocannabinolic acid (THCA) synthase, the enzyme controlling the psychoactivity of Cannabis sativa. J. Mol. Biol. *423*, 96–105.

Thorn, A., Egerer-Sieber, C., Jäger, C.M., Herl, V., Müller-Uri, F., Kreis, W., and Muller, Y.A. (2008). The crystal structure of progesterone 5β-reductase from Digitalis lanata defines a novel class of short chain dehydrogenases/reductases. J. Biol. Chem. *283*, 17260–17269.

Tietze, L.-F. (1983). Iridoids. Part 19. Stereoselective synthesis of iridoid glycosides. Angew. Chem. Int. Ed. Engl. *22*, 828–841.

Tietze, L.-F., and Bartels, C. (1991). Inter- and intramolecular hetero Diels-Alder reactions. 32. Iridoids. 26. Synthesis of bridged homoiridoids from secologanin by tandem Knoevenagel-hetero-Diels-Alder reactions. Liebigs Ann. Chem. *2*, 155–160.

Tietze, L.-F., von Kiedrowski, G., Harms, K., Clegg, W., and Sheldrick, G. (1980). Stereocontrolled intramolecular Diels-Alder reaction of heterodienes; studies on the synthesis of cannabinoids. Angew. Chem. *19*, 130–131.

Tietze, L.-F., von Kiedrowski, G., and Berger, B. (1982). Intramolecular cycloadditions. Part 4. Stereo- and regioselective syntheses of enantiomerically pure (+)- and (-)-hexahydrocannabinol by intramolecular cycloaddition. Angew. Chem. *21*, 222–223.

Tundis, R., Loizzo, M.R., Menichini, F., Statti, G.A., and Menichini, F. (2008). Biological and pharmacological activities of iridoids: recent developments. Mini Rev. Med. Chem. *8*, 399–420.

Uesato, S., Ueda, S., Kobayashi, K., and Inouye, H. (1983). Mechanism of iridane skeleton formation in the biosynthesis of iridoid glucosides in Gardenia jasminoides cell cultures. Chem. Pharm. Bull. (Tokyo) *31*, 4185–4188.

Uesato, S., Matsuda, S., and Inouye, H. (1984). Mechanism for iridane skeleton formation from acyclic monoterpenes in the biosynthesis of secologanin and vindoline in Catharanthus roseus and Lonicera morrowii. Chem. Pharm. Bull. (Tokyo) *32*, 1671–1674.

Uesato, S., Ikeda, H., Fujita, T., Inouye, H., and Zenk, M.H. (1987). Elucidation of iridodial formation mechanism. Partial purification and characterization of the novel monoterpene cyclase from Rauwolfia serpentina cell suspension cultures. Tetrahedron Lett. *28*, 4431–4434.

Vogel, P., Turks, M., Bouchez, L., Marković, D., Varela-Alvarez, A., and Sordo, J.A. (2007). New organic chemistry of sulfur dioxide. Acc. Chem. Res. *40*, 931–942.

Xu, Z., Baunach, M., Ding, L., and Hertweck, C. (2012). Bacterial synthesis of diverse indole terpene alkaloids by an unparalleled cyclization sequence. Angew. Chem. Int. Ed. Engl. *51*, 10293–10297.

Yang, J.W., Hechavarria Fonseca, M.T., and List, B. (2005). Catalytic asymmetric reductive Michael cyclization. J. Am. Chem. Soc. *127*, 15036–15037.

Zhang, W., Ni, C., and Hu, J. (2012). Selective fluoroalkylation of organic compounds by tackling the "negative fluorine effect". Top. Curr. Chem. *308*, 25–44.

Chemistry & Biology, Volume *21*

Supplemental Information

Conversion of Substrate Analogs Suggests a Michael Cyclization in Iridoid Biosynthesis

Stephanie Lindner, Fernando Geu-Flores, Stefan Bräse, Nathaniel H. Sherden, and Sarah E. O'Connor

a

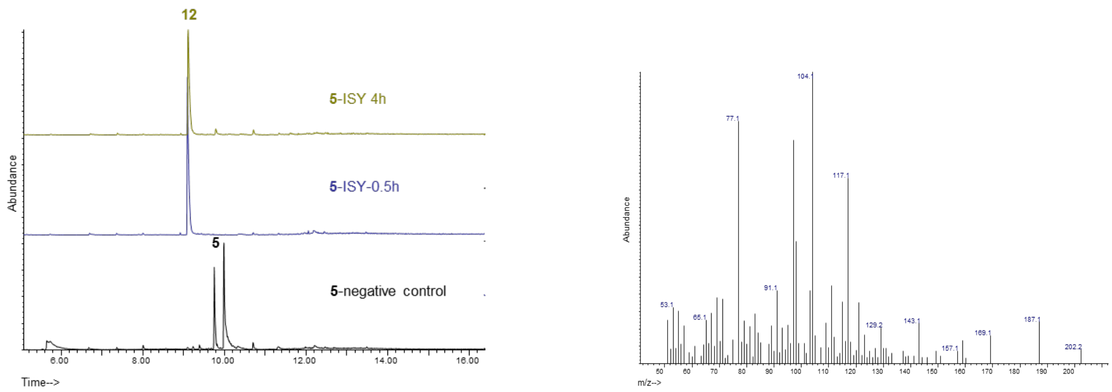
12
5-ISY 4h
5-ISY-0.5h
5
5-negative control
Abundance
Time-->
6.00
8.00
10.00
12.00
14.00
16.00
104.1
77.1
117.1
91.1
53.1
65.1
129.2
143.1
157.1
169.1
187.1
202.2
m/z-->

b

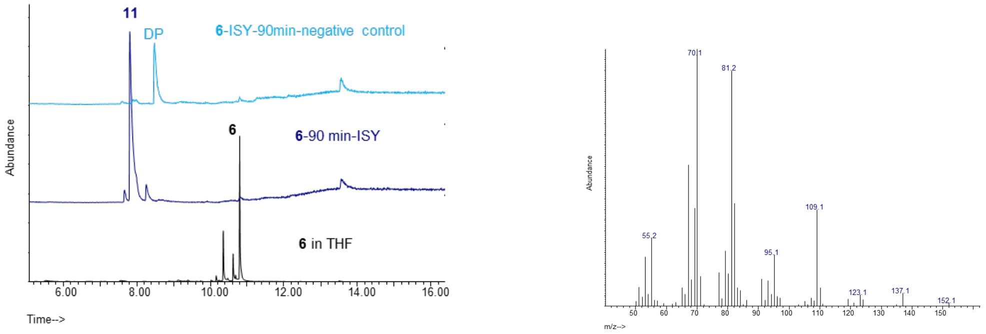
11
DP
6-ISY-90min-negative control
6
6-90 min-ISY
6 in THF
Abundance
Time-->
6.00
8.00
10.00
12.00
14.00
16.00
70.1
81.2
109.1
55.2
95.1
123.1
137.1
152.1
m/z-->

c

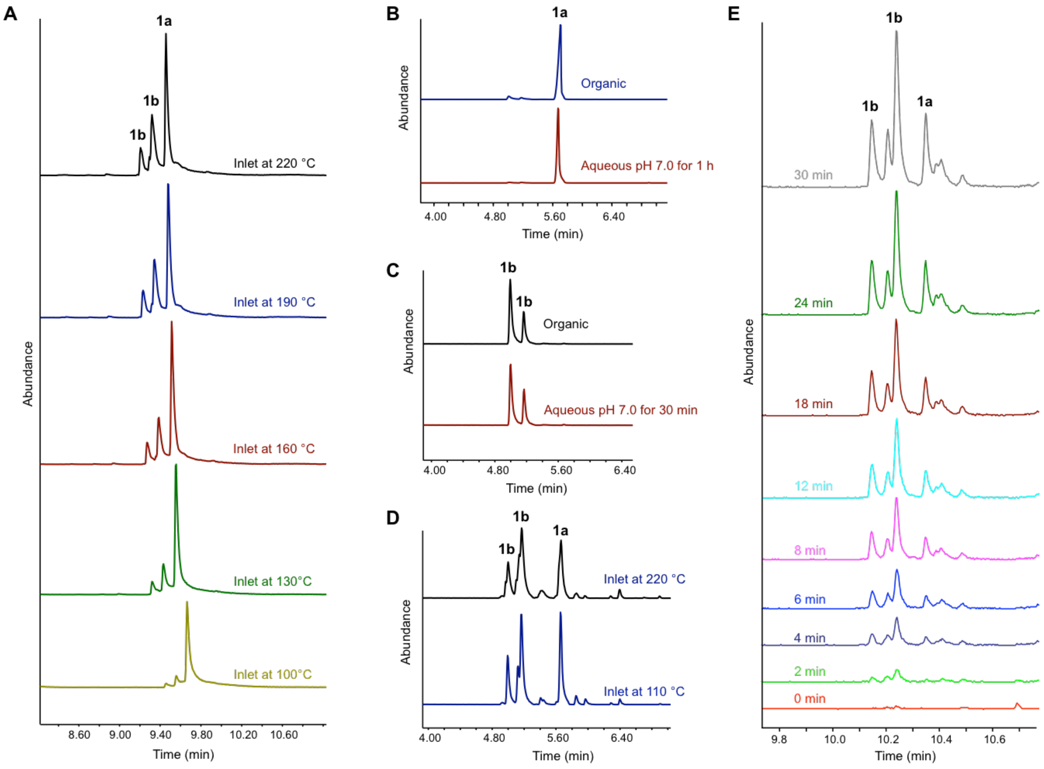
A
1a
1b
1b
Inlet at 220 °C
Inlet at 190 °C
Inlet at 160 °C
Inlet at 130°C
Inlet at 100°C
Abundance
8.60
9.00
9.40
9.80
10.20
10.60
Time (min)
B
1a
Organic
Aqueous pH 7.0 for 1 h
4.00
4.80
5.60
6.40
Time (min)
C
1b
1b
Organic
Aqueous pH 7.0 for 30 min
D
1b
1b
1a
Inlet at 220 °C
Inlet at 110 °C
E
1b
1b
1a
30 min
24 min
18 min
12 min
8 min
6 min
4 min
2 min
0 min
9.8
10.0
10.2
10.4
10.6
Time (min)

Supplementary Fig. 1, related to Figure 1. a. GC-MS chromatograms of iridoid synthase (ISY) with substrate **5**. The mass spectrum of the iridoid synthase product **12** is also shown. **b.** GC-MS chromatograms of iridoid synthase (ISY) with substrate **6**. The mass spectrum of the iridoid synthase product **11** is also shown. DP: Decomposition product. **c.** GC-MS studies on the open (1b) and closed (1b) forms of the iridoid synthase product **1.** For the analysis in panel A, a ZB-5 column was used; for the analysis in panels B-D, a DB-1 column was used; and for the analysis in panel E, an HP-5MS column was used (see details under General Experimental Procedures). (**A**) Pure closed synthetic nepetalactol (**1a**) in CH_2Cl_2 at various inlet temperatures. The amount of open form increases as inlet temperature increases, suggesting substantial thermal opening of the closed form in the inlet at temperatures above 100 °C. At inlet temperatures below 100 °C, separation was compromised (not shown). (**B**) Comparison of synthetic **1a** in CH_2Cl_2 ('organic') and **1a** incubated for 1 hour in neutral aqueous medium (20 mM MOPS, pH 7.0) and then extracted with CH_2Cl_2, using an inlet temperature of 110 °C. No extra peaks appear, indicating that closed form **1a** does not readily equilibrate to the open forms **1b** under the incubation conditions. (**C**) Analysis of the open forms **1b** in CH_2Cl_2 ('organic') or the open forms incubated in neutral aqueous medium (20 mM MOPS, pH 7.0) for 20 minutes and then extracted in CH_2Cl_2, using an inlet temperature of 110 °C. No extra peaks appear, indicating that open forms **1b** do not readily equilibrate to the close form **1a** under the incubation conditions. The open forms **1b** were purified as putative hydrates from the milligram-scale enzymatic assay described in reference 2 in the main text (fractions 5-12 from Column 2; see supplementary Figure 7 of the mentioned publication). (**D**) Product profiles of the ISY reaction at two different inlet temperatures. Both open and closed forms are still present when using the lower inlet temperature (110 °C). (**E**) Time course of the ISY reaction, analyzed using an inlet temperature of 110 °C. The product profile seems virtually identical at all times.

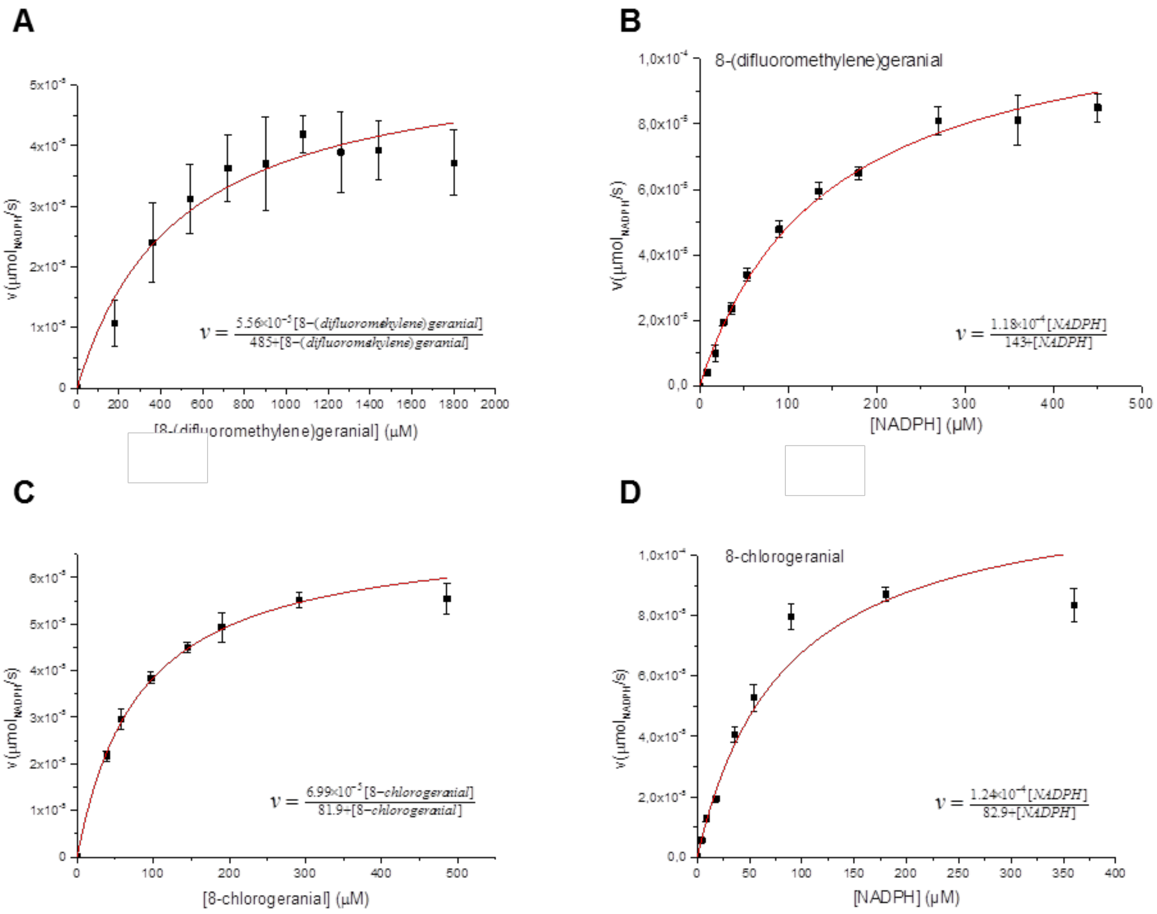

Supplementary Fig. S2, related to Figure 2. Steady-state kinetic analysis of the iridoid synthase reactions. A construct with a truncated N-terminus was used; this truncation has been used for all structural studies of the iridoid synthase homologue, progesterone beta reductase. Reaction rates were measured spectrophotometrically, monitoring NADPH consumption at 340 nm. Individual data points are averages of four replicates. Error bars represent standard errors (SE). The overlaid curves were obtained through non-linear regression (hyperbola), which yielded the rate equations inserted in each plot. (**A**) Saturation curve for 8-(difluoromethylene)geranial at a fixed NADPH concentration of 1 mM. $K_m = 485 \pm 160$ µM; $k_{cat} = 6.4 \pm 0.8$ s^{-1}. (**B**) Saturation curve for NADPH at a fixed 8-(difluoromethylene)geranial concentration of 2 mM. $K_m = 143 \pm 17$ µM; $k_{cat} = 13.6 \pm 1.2$ s^{-1}. (**C**) Saturation curve for 8-chlorogeranial at a fixed NADPH concentration of 160 µM. $K_m = 81.9 \pm 5.6$ µM; $k_{cat} = 8.1 \pm 0.5$ s^{-1}. (**D**) Saturation curve for NADPH at a fixed 8-chlorogeranial concentration of 300 µM. $K_m = 82.9 \pm 14.2$ µM; $k_{cat} = 8.1 \pm 0.5$ s^{-1}. For comparison, the kinetics of this truncated enzyme were measured with native substrate **2**. $K_M = 9.9 \pm 2.1$ µM, $k_{cat} = 1.4 \pm 0.1$ s^{-1}, $k_{cat}/K_M = 0.14$ s^{-1} μM^{-1} (The steady state kinetic parameters of native iridoid synthase with substrate **2** are: $K_M = 4.5 \pm 0.2$ μM, $k_{cat} = 1.6 \pm 0.1$ s^{-1}; for NADPH, $K_M = 4.7 \pm 0.7$ μM, $k_{cat} = 2.2 \pm 0.2$ s^{-1}).

a

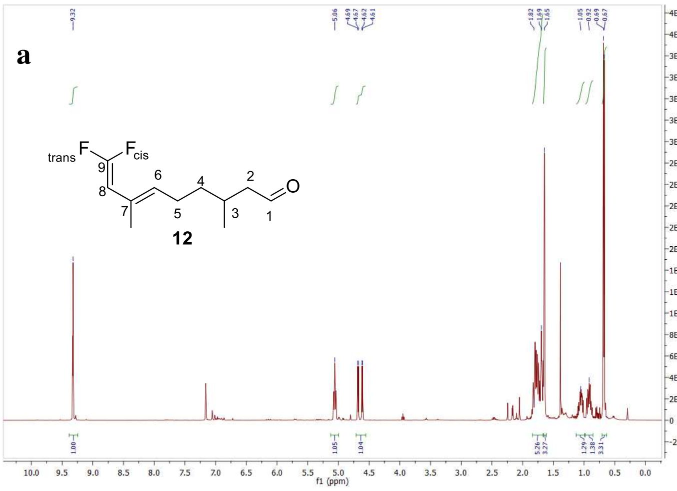

trans F
F cis
12
f1 (ppm)

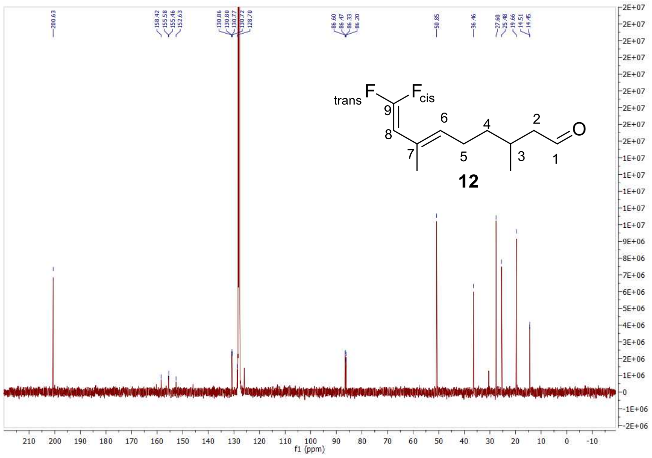

trans F
F cis
12
f1 (ppm)

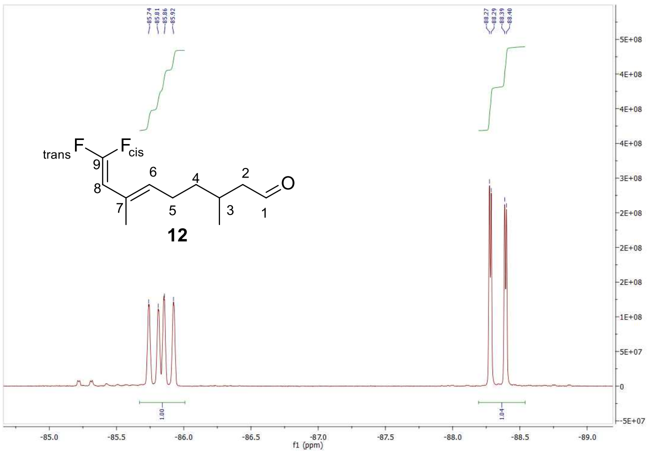

trans F
F cis
12
f1 (ppm)

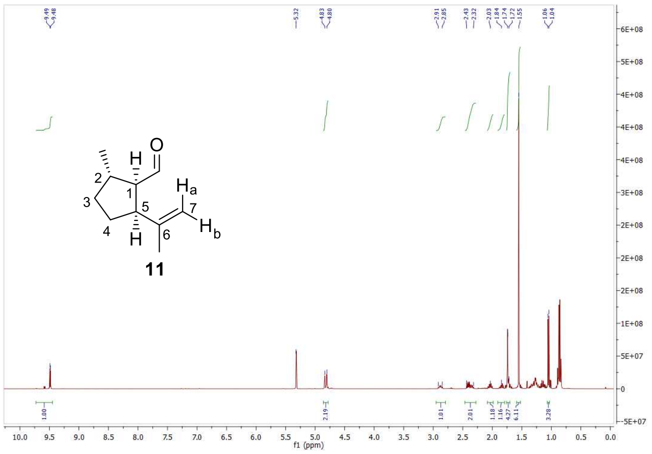

H a
H b
11
f1 (ppm)

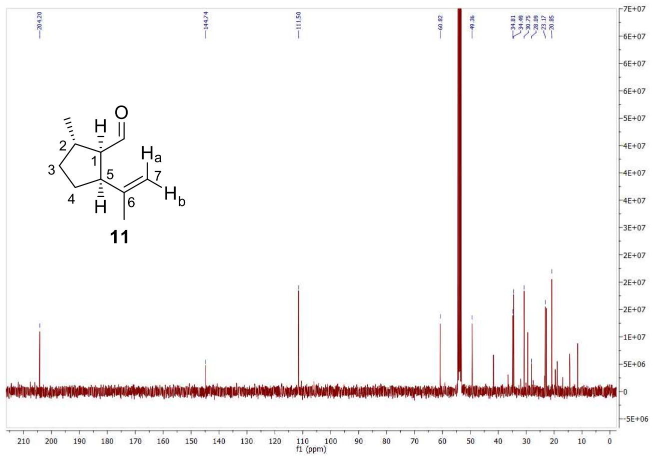

H a
H b
11
f1 (ppm)

b

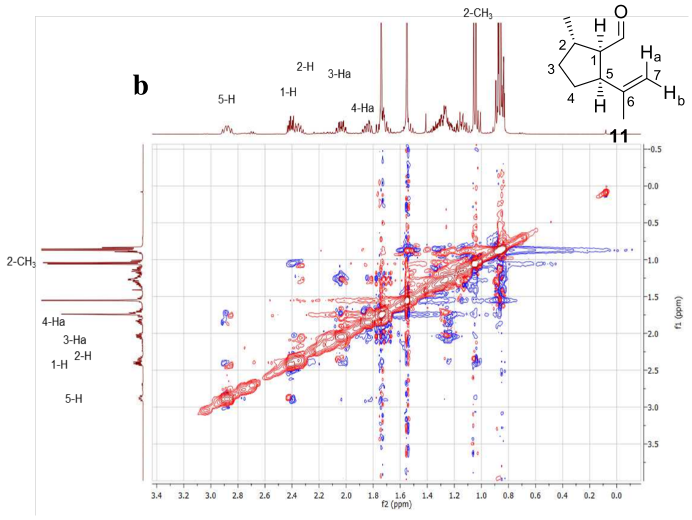

2-CH3
2-H
3-Ha
1-H
5-H
4-Ha
11
f2 (ppm)
f1 (ppm)

Supplementary Fig. S3, related to Figure 3. a. ^{1}H, ^{13}C and ^{19}F NMR spectra of the products resulting from incubation of substrate analogs with iridoid synthase. **b.** ^{1}H, ^{13}C and NOESY NMR spectra of the product resulting from incubation of **6** with iridoid synthase. NOE signals between 1-H and 5-H, and between 2-CH_3 and 1-H suggest the relative stereochemistry drawn.

Supplemental Experimental Procedures

A: General Experimental Procedures

Unless otherwise indicated, all reactions were monitored for completion by Thin Layer Chromatography (TLC). TLC was performed on Merck silica gel 60 F254 precoated (0.25 mm) glass backed plates, 20 x 20 cm, cut down to various sizes for use. TLC results were analyzed by UV fluorescence quenching on fluorescent (F254) plates, or with standard anisaldehyde, cerium sulfate or $KMnO_4$ stains. When necessary, reactions were heated in a silicone oil bath heated and regulated with an IKAmag hot-plate with a temperature-modulating thermocouple. Anhydrous DMF was obtained from Sigma-Aldrich in sure/seal™ bottles and used as received. The NMR solvents $CDCl_3$ and C_6D_6 were purchased from Sigma Aldrich and used as received. CD_2Cl_2 was purchased from Cambridge Isotope Laboratories, Inc. Petroleum ether was obtained from Fisher Scientific and is defined here as petroleum fractions that boil from 36–60 °C. All other standard solvents were purchased from Fisher Scientific and used as received. All filtrations were performed with either fritted glass Büchner funnels or cotton in traditional glass funnels. Activated charcoal (Sigma Aldrich) was used for decolorization. Celite® 545 (Sigma-Aldrich) was used for filtrations. Column chromatography was done using neutral silica gel (particle size D10: 33 µm, D50: 50 µm, D90: 70 µm [≈ 200 – 400 mesh]) from BDH Laboratory Supplies UK or on Florisil® (Sigma Aldrich). All other chemicals used for chemical synthesis were purchased from Sigma Aldrich and used as received.

^{1}H, ^{13}C and ^{19}F NMR spectra were recorded on a Bruker 400 MHz / 54 mm UltraShield Plus, long hold time automated NMR system. ^{1}H spectra are reported relative to residual $CHCl_3$ (at δ 7.26), CH_2Cl_2 (at δ 5.32) or C_6H_6 (at δ 7.16). The description of signals include: s = singlet, d = doublet, t = triplet, q = quartet, sx = sextet, m = multiplet, dd = doublet of doublets, ddd = doublet of dd, dt = doublet of triplets, dq = doublet of quartets. All couplings constants are absolute values.

^{13}C NMR spectra are reported relative to $CDCl_3$ (d = 77.16 ppm), CD_2Cl_2 (d = 53.84 ppm) and C_6D_6 (d = 128.06 ppm). The assignment of the atoms was made via partial characterization by 1D (^{1}H, ^{13}C, DEPT) and 2D (COSY, NOESY, HSQC) NMR. The signal structure was analyzed by DEPT and is described as follows: + = primary or tertiary C-atom (positive signal), – = secondary C-atom (negative signal), and $C_{quart.}$ = quaternary C-atom (no signal).

Standard GC-MS spectra were recorded on an Agilent 6890N GC system equipped with a split/splitless injector and coupled to an Agilent 5973 MS detector. Helium was used as carrier gas at 1 mL/min. The temperature of the inlet was 220 °C unless otherwise specified. For the analysis of the synthesized substrate analogs and their enzymatic products, separations were carried out with a Zebron ZB-5 HT column (30 m × 0.25 mm × 0.10 µm) using the following program: 5 min isothermal at 60 °C, then 20 °C/min gradient up to 150 °C, then 45°C/min gradient up to 280 °C (run time = 16.39 min). For the analysis of the open/closed forms of the native enzymatic product, separations were carried out either with the mentioned ZB-5 HT column and program or with an Agilent DB-1ht column (15 m × 0.25 mm × 0.10 µm) using the following program: 2 min isothermal at 60 °C, then 12 °C/min gradient up to 150 °C, then 45°C/min gradient up to 280 °C (run time = 14.39 min). For the reaction time course, separations were carried out using an Agilent HP-5MS column (30 m x 0.25 mm x 0.25 µm) and the same temperature program as the one used for the ZB-5 HT column (see above).

GC-MS based accurate mass determination was performed on a Waters GCT system consisting of an Agilent 6890 Series GC system fitted with split/splitless injector and coupled to a Waters GCT Classic Mass Spectrometer at Rothamsted

Research. In case where no accurate mass spectra could be measured due to the instability of the compound, the standard GC-MS is provided for characterization.

B: Synthesis of the test substrates

Synthesis of 8-oxogeranyl acetal ***16***

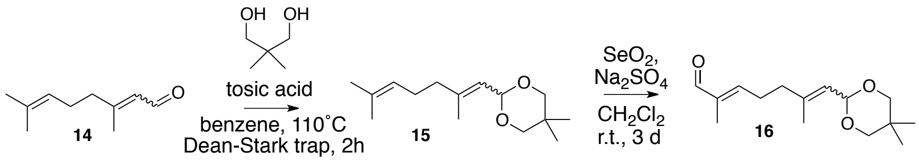

a) Synthesis of geranyl acetal [2-(2,6-dimethylhepta-1,5-dien-1-yl)-5,5-dimethyl-1,3-dioxane, **15**]:

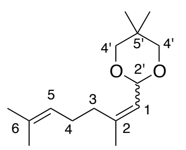

Tosic acid monohydrate (125 mg, 0.657 mmol, 0.01 equiv.) was added to a mixture of citral (10.0 g, 65.7 mmol, 1.00 equiv.) and 2,2-dimethylpropane-1,3-diol (20.5 g, 197 mmol, 3.00 equiv.) in benzene (100 mL). The flask was connected to a Dean-Stark trap and the mixture was refluxed at 110 °C for 2 h. After cooling to r.t. the reaction mixture was washed with 10% $NaHCO_{3(aq)}$ (3 × 150 mL) and the aqueous layers were back extracted with diethyl ether (100 mL). The combined organic phases were washed with brine (150 mL), dried over sodium sulfate, filtered and concentrated *in vacuo* affording crude **14** as a yellow oil in quantitative yield. The crude geranyl acetate product was used in subsequent reaction without farther purification. See I. Grosu, S. Mager, I. Batiu, *Rev. Roum. Chem.* **1995**, *40*, 1175-1182 for a prior synthesis.

^{1}H NMR (400 MHz, $CDCl_3$): δ = 5.34 (dd, ^3J = 6.3 Hz, ^4J = 1.1 Hz, 1 H, 1-H), 5.16–5.04 (m, 2 H, 5-H, 2'-H), 3.67–3.60 (m, 2 H, 4'-H_a), 3.54–3.46 (m, 2 H, 4'-H_b), 2.18–1.96 (m, 4 H, 3-H, 4-H), 1.76 (d, ^4J = 1.4 Hz, 3 H, 2_Z-CH_3), 1.74 (d, ^4J = 1.2 Hz, 3 H, 2_E-CH_3), 1.69 (s, 3 H, 6_{Za}-CH_3), 1.67 (s, 3 H, 6_{Ea}-CH_3), 1.61 (s, 3 H, 6_{Zb}-CH_3), 1.59 (s, 3 H, 6_{Eb}-CH_3), 1.22 (s, 3 H, $5'_{Ea}$-CH_3), 1.21 (s, 3 H, $5'_{Za}$-CH_3), 0.74 (s, 3 H, $5'_{Eb}$-CH_3), 0.73 (s, 3 H, $5'_{Zb}$-CH_3) ppm.

b) Synthesis of 8-oxogeranyl acetal [(2*E*,6*E*/*Z*)-7-(5,5-dimethyl-1,3-dioxan-2-yl)-2,6-dimethylhepta-2,6-dienal, **16**]:

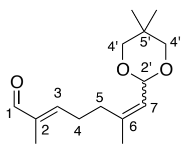

Crude geranyl acetal **15** (10.0 g, 42.0 mmol, 1.00 equiv.) was added to a mixture of SeO_2 (5.13 g, 46.2 mmol, 1.10 equiv.) and Na_2SO_4 (11.9 g, 84.0 mmol, 2.00 equiv.) in dichloromethane (100 mL) under nitrogen. The reaction mixture was stirred for three days at room temperature and then filtered through a thin pad of Celite® rinsing with dichloromethane. After concentrating the solution to roughly one-third of its original volume *in vacuo*, a 1:1 mixture of hexanes/ethyl acetate (200 mL) was added. The mixture was washed with 1:6:9 NaCl/bleach/H_2O (3 × 150 mL), saturated $NaHCO_{3(aq)}$ solution (3 × 150 mL), a 60 mg/mL solution of $Na_2SO_{3(aq)}$ (3 × 150 mL) and brine (2 × 150 mL). The organic phase was simultaneously dried and decolorized by stirring with a mixture of sodium sulfate and activated charcoal for one hour. The resulting black suspension was filtered through a thin pad of Celite® rinsing with ethyl acetate and concentrated *in vacuo*. Chromatography on silica (hexanes/EtOAc = 20/1 to 10/1) afforded 8-oxogeranyl acetal **16** as a separable mixture of two diastereomers (6*Z*:6*E* ≈ 1:1.5) in 11% yield (1.21 g, 4.77 mmol) as a yellow oil. The product could not be purified completely, and was partially still bound to selenium (seen by GC-MS).

6*Z*-isomer: $\boldsymbol{R}_f$ = 0.38 (hexanes/ethyl acetate = 5/1). **^{1}H NMR** (400 MHz, $CDCl_3$): δ = 9.39 (s, 1 H, 1-H), 6.54–6.48 (m, 1 H, 3-H), 5.42 (d, ^3J = 6.4 Hz, 1 H, 7-H), 5.03 (d, ^3J = 6.4 Hz, 1 H, 2'-H), 3.63 (dd, ^2J = 10.5 Hz, ^4J = 1.3 Hz, 2 H, 4'-H_a), 3.47 (d, ^2J = 10.5 Hz, 2 H, 4'-H_b), 2.52–2.26 (m, 4 H, 4-H, 5-H), 1.79 (d, ^4J = 1.5 Hz, 3 H, 6-CH_3), 1.76 (d, ^4J = 1.1 Hz, 3 H, 2-CH_3), 1.21 (s, 3 H, 5'-CH_{3a}), 0.73 (s, 3 H, 5'-CH_{3b}) ppm. **^{13}C NMR** (100 MHz, $CDCl_3$): δ = 195.4 (+, C-1), 153.6 (+, C-3), 141.6 (C_{quart}, C-2), 139.8 (C_{quart}, C-6), 124.5 (+, C-7), 98.5 (+, C-2'), 77.4 (–, C-4'$_a$), 77.2 (–, C-4'$_b$), 31.5 (–, C-5), 30.1 (C_{quart}, C-5'), 27.4 (–, C-4), 23.1 (+, 5'-CH_{3a}), 23.1 (+, 5'-CH_{3b}), 22.8 (+, 6-CH_3), 9.3 (+, 2-CH_3) ppm. The signal of C-4'$_b$ at 77.2 ppm is hidden by the solvent signal but could be determined via HSQC. **6*E*-isomer**: $\boldsymbol{R}_f$ = 0.33 (hexanes/ethyl acetate = 5/1). **^{1}H NMR** (400 MHz, $CDCl_3$): δ = 9.38 (s, 1 H, 1-H), 6.46 (tq, ^3J = 7.1 Hz, ^4J = 1.4 Hz, 1 H, 3-H), 5.37 (dq, ^3J = 6.3 Hz, ^4J = 1.3 Hz, 1 H, 7-H), 5.09 (d, ^3J = 6.3 Hz, 1 H, 2'-H), 3.67–3.44 (m, 4 H, 4'-H), 2.50 (q, ^3J = 7.4 Hz, 2 H, 4-H), 2.21 (t, ^3J = 7.8 Hz, 2 H, 5-H), 1.78 (d, ^4J = 1.1 Hz, 3 H, 6-CH_3), 1.74 (d, ^4J = 1.4 Hz, 3 H, 2-CH_3), 1.22 (s, 3 H, 5'-CH_{3a}), 0.74 (s, 3 H, 5'-CH_{3b}) ppm. **^{13}C NMR** (100 MHz, $CDCl_3$): δ = 195.3 (+, C-1), 153.6 (+, C-3), 141.3 (C_{quart}, C-2), 139.8 (C_{quart}, C-6), 123.1 (+, C-7), 98.8 (+, C-2'), 37.5 (–, C-5), 30.2 (C_{quart}, C-5'), 27.0 (–, C-4), 23.1 (+, 5'-CH_{3a}), 22.1 (+, 5'-CH_{3b}), 17.4 (+, 6-CH_3), 9.3 (+, 2-CH_3) ppm. The two signals of C-4' are hidden by the solvent signal and could not be determined.

Synthesis of the Diels-Alder test substrate 8-(difluoromethylene)geranial **5**

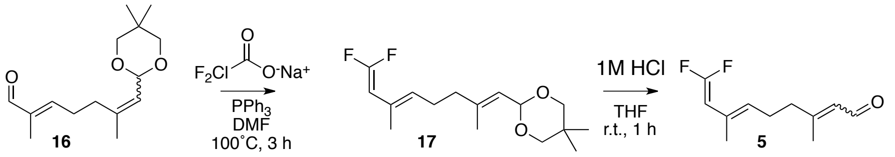

a) Synthesis of 8-(difluoromethylene)geranyl acetal [2-((1*E*/*Z*,5*E*)-8,8-difluoro-2,6-dimethylocta-1,5,7-trien-1-yl)-5,5-dimethyl-1,3-dioxane, **17**]:

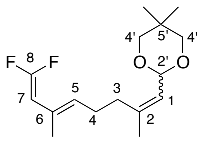

A solution of 8-oxogeranyl acetal **16** (mixture of both diastereomers, 400 mg, 1.59 mmol, 1.00 equiv.) in absolute DMF (1 mL) under nitrogen was added to a mixture of sodium chlorodifluoroacetate (725 mg, 4.76 mmol, 3.00 equiv.) and triphenylphosphine (1.25 g, 4.76 mmol, 3.00 equiv.) in absolute DMF (1 mL) under nitrogen at 30–40 °C. The reaction mixture was immediately heated up to 100 °C and stirred for 3 h at 100–105 °C, turning dark brown. After cooling to room temperature, diethyl ether (10 mL) and water (10 mL) were added. The resulting mixture was filtered, followed by rinsing with copious amounts of diethyl ether. The phases were separated and the aqueous phase was extracted with diethyl ether (3 × 10 mL). The combined organic phases were washed with brine (2 × 20 mL), dried over sodium sulfate, filtered and concentrated *in vacuo*. Chromatography on silica (hexanes/EtOAc = 50/1 to 25/1) afforded 8-(difluoromethylene)geranyl acetal **17** in 18% yield (79.8 mg, 0.279 mmol) as yellow oil. The reaction was also implemented using only the *6Z*-isomer or the 6*E*-isomer of starting material **15**, respectively. Thus the diastereomers of **16** could be obtained separately.

1*Z*-isomer: $\boldsymbol{R}_f$ = 0.61 (hexanes/ethyl acetate = 5/1). **^{1}H NMR** (400 MHz, CD_2Cl_2): δ = 5.39 (t, ^3J = 7.0 Hz, 1 H, 5-H), 5.28–5.24 (m, 1 H, 1-H), 5.00 (d, ^3J = 6.4 Hz, 1 H, 2'-H), 4.89 (dd, $^3J_{HF}$ = 5.3, 26.9 Hz, 1 H, 7-H), 3.57 (d, ^2J = 10.5 Hz, 2 H, 4'-H_a), 3.45 (d, ^2J = 10.5 Hz, 2 H, 4'-H_b), 2.24–2.09 (m, 4 H, 3-H, 4-H), 1.83–1.81 (m, 3 H, 6-CH_3), 1.74 (d, 3 H, ^4J = 1.5 Hz, 2-CH_3), 1.16 (s, 3 H, 5'-CH_{3a}), 0.71 (s, 3 H, 5'-CH_{3b}) ppm. **^{13}C NMR** (100 MHz, CD_2Cl_2): δ = 142.0 (C_{quart}, C-2), 130.5 (C_{quart}, dd, $^3J_{CF}$ = 5.9, 8.8 Hz, C-6), 126.6 (m, C-2'), 124.1 (+, C-5), 98.9 (+, C-1), 86.3 (+, dd, $^2J_{CF}$ = 12.5, 27.1 Hz, C-7), 77.5 (–, 2 × C-4'), 32.7 (–, C-3), 30.1 (C_{quart}, C-5'), 26.8 (–, C-4), 23.2 (+, 5'-CH_{3a}), 23.1 (+, 5'-CH_{3b}), 22.0 (+, 2-CH_3), 14,6 (+, d,

$^4J_{CF}$ = 5.9 Hz, 6-CH_3) ppm. The signal of C-8 could not be detected, presumably because of low sample amount. **^{19}F NMR** (376 MHz, CD_2Cl_2): δ = –85.9 (ddd, $^2J_{FF}$ = 42.9 Hz, $^3J_{FH}$ = 26.6 Hz, $^5J_{FH}$ = 3.0 Hz, 1 F, F_{cis}), –85.24 (dd, $^2J_{FF}$ = 42.9 Hz, $^3J_{FH}$ = 3.2 Hz, 1 F, F_{trans}) ppm. **1*E*-isomer**: ***R*** R_f = 0.61 (hexanes/ethyl acetate = 5/1). **^{1}H NMR** (400 MHz, CD_2Cl_2): δ = 5.39 (t, ^3J = 7.0 Hz, 1 H, 5-H), 5.27–5.23 (m, 1 H, 1-H), 5.02 (d, ^3J = 6.3 Hz, 1 H, 2'-H), 4.89 (dd, $^3J_{HF}$ = 5.4, 27.0 Hz, 1 H, 7-H), 3.57 (d, ^2J = 11.2 Hz, 2 H, 4'-H_a), 3.47 (d, ^2J = 11.0 Hz, 2 H, 4'-H_b), 2.25–2.02 (m, 4 H, 3-H, 4-H), 1.82–1.79 (m, 3 H, 6-CH_3), 1.71 (d, 3 H, ^4J = 1.4 Hz, 2-CH_3), 1.16 (s, 3 H, 5'-CH_{3a}), 0.71 (s, 3 H, 5'-CH_{3b}) ppm. **^{13}C NMR** (100 MHz, CD_2Cl_2): δ = 141.9 (C_{quart}, C-2), 130.5 (C_{quart}, dd, $^3J_{CF}$ = 5.9, 8.8 Hz, C-6), 126.6 (m, C-2'), 123.1 (+, C-5), 99.2 (+, C-1), 86.3 (+, dd, $^2J_{CF}$ = 12.5, 27.1 Hz, C-7), 77.5 (–, 2 × C-4'), 39.0 (–, C-3), 30.2 (C_{quart}, C-5'), 26.4 (–, C-4), 23.1 (+, 5'-CH_{3a}), 22.0 (+, 5'-CH_{3b}), 17.2 (+, 2-CH_3), 14.6 (+, d, $^4J_{CF}$ = 5.9 Hz, 6-CH_3) ppm. The signal of C-8 could not be detected. **^{19}F NMR** (376 MHz, CD_2Cl_2): δ = –85.8–(–86.1) (ddd, m, 1 F, F_{cis}), –88.9 (dd, $^2J_{FF}$ = 43.7 Hz, $^3J_{FH}$ = 5.5 Hz, 1 F, F_{trans}) ppm.

b) Synthesis of 8-(difluoromethylene)geranial [(6*E*)-9,9-difluoro-3,7-dimethylnona-2,6,8-trienal, **5**]:

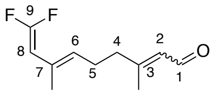

$HCl_{(aq)}$ (1 M, 1 mL) was added to a solution of 8-(difluoromethylene)geranyl acetal **17** (79.8 mg, 0.279 mmol, 1.00 equiv.) in tetrahydrofuran (2 mL). After stirring at room temperature for 1 h, a saturated solution of $NaHCO_{3(aq)}$ (2 mL) was carefully added and the mixture was extracted with ethyl acetate (3 × 5 mL). The merged organic phases were washed with $NaHCO_{3(aq)}$ (5 mL), brine (5 mL), dried over sodium sulfate, filtered and concentrated *in vacuo*. Chromatography on silica (hexanes/EtOAc = 10/1) afforded 8-(difluoromethylene)geranial **5** as an inseparable mixture of two diastereomers in 71% yield (39.6 mg, 0.198 mmol) as a yellow oil. The reaction was also implemented using only the 1*Z*-isomer or the 1*E*-isomer of starting material **17**; however, these reactions also formed a mixture of both diastereomers due to rapid isomerization. Compound **5** is quite reactive and is best stored in solution (e.g. tetrahydrofuran) at –20 °C.

R_f = 0.46 (*Z*), 0.41 (*E*) (hexane/ethyl acetate = 5/1). **^{1}H NMR** (400 MHz, C_6D_6): both diastereomers: δ = 9.86 (d, ^3J = 7.8 Hz, 1 H, $CHO_{(E)}$), 9.82 (d, ^3J = 7.7 Hz, 1 H, $CHO_{(Z)}$), 5.79–5.73 (m, 2 H, 2-$H_{(E/Z)}$), 4.93–4.85 (m, 2 H, 6-$H_{(E/Z)}$), 4.61–4.50 (m, 2 H, 8-$H_{(E/Z)}$), 2.00 (t, ^3J = 7.5 Hz, 2 H, 4-$H_{(Z)}$), 1.81–1.58 (m, 6 H, 4-$H_{(E)}$, 4,5-$H_{(E/Z)}$), 1.57–1.54 (m, 3 H, 7-$CH_{3(E)}$), 1.53–1.50 (m, 3 H, 7-$CH_{3(Z)}$), 1.45 (s, 3 H, 3-$CH_{3(E)}$), 1.32 (s, 3 H, 3-$CH_{3(Z)}$) ppm. **^{13}C NMR** (100 MHz, C_6D_6): both diastereomers: δ = 189.6 (+, C-$1_{(E)}$), 189.1 (+, C-$1_{(Z)}$), 160.7 (C_{quart}, C-$3_{(Z)}$), 160.6 (C_{quart}, C-$3_{(E)}$), 155.6 (C_{quart}, dd, $^1J_{CF}$ = 297.8, 286.1 Hz, C-$9_{(Z)}$), 155.6 (C_{quart}, dd, $^1J_{CF}$ = 297.1, 285.4 Hz, C-$9_{(E)}$), 129.2–126.5 (C-$2_{(E/Z)}$, C-$6_{(E/Z)}$, C-$7_{(E/Z)}$), 86.1 (+, dd, $^3J_{CF}$ = 27.5, 12.0 Hz, C-$8_{(E)}$), 86.0 (+, dd, $^3J_{HF}$ = 27.5, 12.0 Hz, C-$8_{(Z)}$), 39.7 (–, C-$4_{(E)}$), 31.8 (–, C-$4_{(Z)}$), 26.8 (–, C-$5_{(Z)}$), 25.5 (–, C-$5_{(E)}$), 24.3 (+, 3-$CH_{3(Z)}$), 16.8 (+, 3-$CH_{3(E)}$), 14.4 (+, d, $^4J_{CF}$ = 5.8 Hz, 7-$CH_{3(E/Z)}$) ppm. The signals of C-2 and C-6 could only be detected *via* HSQC and not be unambiguously determined, because they are overlapped by the solvent signal (C_6D_6). C-7 could not be detected, but is also likely overlapped by the solvent signal. **^{19}F NMR** (376 MHz, C_6D_6): both diastereomers: δ = –84.95 (dd, $^2J_{FF}$ = 41.1 Hz, $^3J_{FH}$ = 26.2 Hz, 1 F, *cis*-$F_{(Z)}$), –85.24 (dd, $^2J_{FF}$ = 40.7 Hz, $^3J_{FH}$ = 27.0 Hz, 1 F, *cis*-$F_{(E)}$), –87.31–(–87.46) (m, 1 H, *trans*-$F_{(Z)}$), –87.61–(–87.77) (m, 1 H, *trans*-$F_{(E)}$) ppm. HR-GCMS calculated for $C_{11}H_{14}F_2O$ $[M]^+$: 200.1013, found 200.1019 (*Z*), 200.1015 (*E*).

Synthesis of the Michael addition test substrate 8-chlorogeranial ***6***

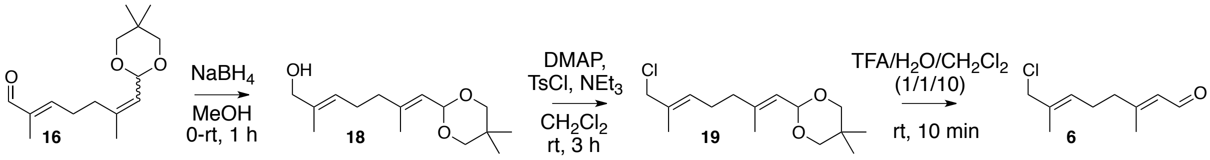

a) Synthesis of 8-hydroxygeranyl acetal [(2*E*,6*E*/*Z*)-7-(5,5-dimethyl-1,3-dioxan-2-yl)-2,6-dimethylhepta-2,6-dien-1-ol, **18**]:

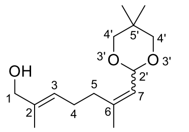

Sodium borohydride (122 mg, 3.23 mmol, 2.00 equiv.) was added to a solution of 8-oxogeranyl acetal **16** (408 mmol, 1.61 mmol, 1.00 equiv.) in methanol (8 mL) at 0 °C, turning the mixture light yellow, then red. The reaction mixture was allowed to warm up to room temperature where stirring was continued for 1.5 h. Additional sodium borohydride (122 mg, 3.23 mmol, 2.00 equiv.) was added and the mixture was stirred for another 10 minutes. Saturated $NaHCO_{3(aq)}$ (8 mL) was added slowly and the reaction mixture was extracted with dichloromethane (3 × 10 mL). The combined organic layers were washed with brine (2 × 10 mL), dried over sodium sulfate, filtered and concentrated *in vacuo*. Chromatography on silica (hexanes/EtOAc = 5/1) afforded 8-hydroxygeranyl acetal **18** as a mixture of two diastereomers in 36% yield (146 mg, 0.574 mmol) as yellow oil. The reaction was also implemented using only the 6*Z*-isomer or the 6*E*-isomer of starting material **16**, respectively. Thus the diastereomers of **18** could be obtained separately. It should be noted, that the starting material bound to selenium didn't react.

***6Z*-isomer**: $\boldsymbol{R}_\mathbf{f}$ = 0.40 (hexanes/ethyl acetate = 2/1). **^{1}H NMR** (400 MHz, CD_2Cl_2): δ = 5.39–5.33 (m, 1 H, 3-H), 5.28–5.24 (m, 1 H, 7-H), 5.00 (d, ^3J = 6.9 Hz, 1 H, 2'-H), 3.92 (d, ^3J = 5.3 Hz, 2 H, 1-H), 3.58 (dd, ^2J = 9.9 Hz, ^4J = 1.3 Hz, 2 H, 4'-H_a), 3.45 (dd, ^2J = 11.2 Hz, ^4J = 0.8 Hz, 2 H, 4'-H_b), 2.18–2.14 (m, 4 H, 4-H, 5-H), 1.75 (d, ^4J = 1.5 Hz, 3 H, 6-CH_3), 1.63 (d, ^4J = 1.3 Hz, 3 H, 2-CH_3), 1.16 (s, 3 H, 5'-CH_{3a}), 0.71 (s, 3 H, 5'-CH_{3b}) ppm. **^{13}C NMR** (100 MHz, CD_2Cl_2): δ = 141.8 (C_{quart}, C-6), 136.4 (C_{quart}, C-2), 125.1 (+, C-5), 123.9 (+, C-7), 99.0 (+, C-2'), 77.4 (–, 2 × C-4'), 69.0 (–, C-1), 32.7 (–, C-5), 30.1 (C_{quart}, C-5'), 26.0 (–, C-4), 23.2 (+, 5'-CH_{3a}), 23.0 (+, 5'-CH_{3b}), 22.0 (+, 6-CH_3), 13.8 (+, 2-CH_3) ppm. ***6E*-isomer**: $\boldsymbol{R}_\mathbf{f}$ = 0.36 (hexanes/ethyl acetate = 2/1). **^{1}H NMR** (400 MHz, CD_2Cl_2): δ = 5.35 (sxd, ^3J = 7.0 Hz, ^4J = 1.3 Hz, 1 H, 3-H), 5.23 (sxd, ^3J = 6.4 Hz, ^4J = 1.3 Hz, 1 H, 7-H), 5.04 (d, ^3J = 6.4 Hz, 1 H, 2'-H), 3.94 (s, 2 H, 1-H), 3.58 (dd, ^2J = 9.9 Hz, ^4J = 1.3 Hz, 2 H, 4'-H_a), 3.47 (dd, ^2J = 11.2 Hz, ^4J = 0.8 Hz, 2 H, 4'-H_b), 2.21–2.03 (m, 4 H, 4-H, 5-H), 1.71 (d, ^4J = 1.3 Hz, 3 H, 6-CH_3), 1.64 (s, 3 H, 2-CH_3), 1.17 (s, 3 H, 5'-CH_{3a}), 0.71 (s, 3 H, 5'-CH_{3b}) ppm. **^{13}C NMR** (100 MHz, CD_2Cl_2): δ = 141.9 (C_{quart}, C-6), 136.0 (C_{quart}, C-2), 125.4 (+, C-5), 123.2 (+, C-7), 99.2 (+, C-2'), 77.5 (–, 2 × C-4'), 69.1 (–, C-1), 39.1 (–, C-5), 30.2 (C_{quart}, C-5'), 25.9 (–, C-4), 23.1 (+, 5'-CH_{3a}), 22.0 (+, 5'-CH_{3b}), 17.1 (+, 6-CH_3), 14.2 (+, 2-CH_3) ppm.

b) Synthesis of 8-chlorogeranyl acetal [2-((1*E*,5*E*)-7-chloro-2,6-dimethylhepta-1,5-dien-1-yl)-5,5-dimethyl-1,3-dioxane, **19**]:

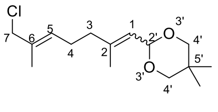

DMAP (43.9 mg, 0.359 mmol, 0.625 equiv.), tosyl chloride (131 mg, 0.689 mmol, 1.20 equiv.) and NEt_3 (80 µL, 58.1 mg, 0.574 mmol, 1.00 equiv.) were sequentially added to a solution of 8-hydroxygeranyl acetal **18** (146 mg, 0.574 mmol, 1.00 equiv.) in dichloromethane (3 mL) under nitrogen. After stirring for 3 h at room temperature, the solvent was removed *in vacuo*. Chromatography on Florisil® (hexanes/EtOAc = 10/1 + 5% NEt_3) afforded 8-chlorogeranyl acetal **19** as an inseparable mixture of two diastereomers in 45% yield (69.7 mg, 0.255 mmol) as yellow oil. The reaction was also implemented using only the 6*E*-isomer of starting material **18**. Thus the 1*E*-diastereomer of **19** could be obtained analytically pure.

$\boldsymbol{R}_\mathbf{f}$ = 0.64 (hexanes/ethyl acetate = 10/1). ***1E*-isomer**: **^{1}H NMR** (400 MHz, CD_2Cl_2): δ = 5.54 (dt, ^3J = 7.0 Hz, ^4J = 1.1 Hz, 1 H, 5-H), 5.25 (sxd, ^3J = 6.3 Hz, ^4J = 1.3 Hz 1 H, 1-H), 5.03 (d, ^3J = 6.3 Hz, 1 H, 2'-H), 4.02 (s, 2 H, 7-H), 3.57 (d,

^2J = 11.2 Hz, 2 H, 4'-H_a), 3.47 (d, ^2J = 10.9 Hz, 4'-H_b), 2.21–2.02 (m, 4 H, 3-H, 4-H), 1.72 (d, ^4J = 1.1 Hz, 3 H, 6-CH_3), 1.70 (d, ^4J = 1.3 Hz, 3 H, 2-CH_3), 1.15 (s, 3 H, 5'-CH_{3a}), 0.71 (s, 3 H, 5'-CH_{3b}) ppm. **^{13}C NMR** (100 MHz, CD_2Cl_2): δ = 141.83 (C_{quart}, C-2), 132.6 (C_{quart}, C-6), 130.4 (+, C-5), 123.1 (+, C-1), 99.2 (+, C-2'), 77.5 (–, 2 × C-4'), 52.9 (–, C-7), 38.7 (–, C-3), 30.2 (C_{quart}, C-5'), 26.6 (–, C-4), 23.1 (+, 5'-CH_{3a}), 22.0 (+, 5'-CH_{3b}), 17.2 (+, 2-CH_3), 14.2 (+, 6-CH_3) ppm.

c) Synthesis of 8-chlorogeranial [(6*E*,2*E*/*Z*)-8-chloro-3,7-dimethylocta-2,6-dienal, **6**]: A mixture of TFA/H_2O (1:1, 0.4 mL)

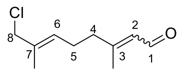

was added to a solution of 8-chlorogeranyl acetal **19** (69.7 mg, 0.255 mmol, 1.00 equiv.) in dichloromethane (2 mL). After stirring for 10 minutes, saturated $NaHCO_{3(aq)}$ (2 mL) was added to the reaction carefully. The mixture was immediately extracted with dichloromethane (3 × 5 mL), the combined organic layers were dried over sodium sulfate, filtered and concentrated *in vacuo*. Chromatography on Florisil® (hexanes/EtOAc = 10/1 + 5% NEt_3) afforded 8-chlorogeranial **6** as an inseparable mixture of two diastereomers in 69% yield (32.7 mg, 0.175 mmol) as yellow oil. The reaction was also implemented using only the 1*E*-isomer of starting material **6**, also leading to a mixture of both diastereomers due to rapid isomerization. The molecule reacted easily, resulting in the rearranged product with a mass of 168.1, which was obtained as an inseparable byproduct.

***R*f** = 0.28 (hexanes/ethyl acetate = 10/1). **Major isomer (2*E*): ^{1}H NMR** (400 MHz, CD_2Cl_2): δ = 9.98 (d, ^3J = 8.0 Hz, 1 H, 1-H), 5.83 (dd, ^3J = 8.0 Hz, ^4J = 1.2 Hz, 1 H, 2-H), 5.58–5.48 (m, 1 H, 6-H), 4.02 (s, 2 H, 8-H), 2.29–2.25 (m, 4 H, 4-H, 5-H), 2.16 (d, ^4J = 1.2 Hz, 3 H, 3-CH_3), 1.75–1.73 (m, 3 H, 7-CH_3) ppm. **^{13}C NMR** (100 MHz, CD_2Cl_2): δ = 191.3 (+, C-1), 163.2 (C_{quart}, C-3), 133.5 (C_{quart}, C-7), 129.2 (+, C-6), 127.8 (+, C-2), 52.5 (–, C-8), 40.0 (–, C-4), 26.0 (–, C-5), 17.7 (+, 3-CH_3), 14.3 (+, 7-CH_3) ppm. HR-GCMS calculated for $C_9H_{12}O$ $[M-CH_3]^+$: 171.0577, found 171.0585 (*Z*), 171.0577 (*E*).

C: Enzymatic assays

All enzyme assays were carried out using 20 mM MOPS pH 7.0 as buffer. The substrates were kept as 50 mM stocks in tetrahydrofuran (THF) at −20 °C. During the preparation of master mixes, care was taken not to exceed THF concentrations higher than 0.5% in the presence of enzyme, as concentrations above 1% THF were found to affect activity adversely. The batch of THF used contained a small amount of inhibitor of peroxide formation (butylated hydroxytoluene, BHT, added by the solvent manufacturer), which is visible as a small peak at approximately 10.35 min in the GC chromatograms of extracted enzymatic products.

Cloning and expression of truncated iridoid synthase

The USER-compatible vector pET28au was created by amplifying the GFP coding sequence from pEAQ-*HT*-GFP [Sainsbury et al. (2011) *Plant Biotechnol J* **7**:682] using primers **AAAAAAGGATCCGCTGAGGCCTTAATTAAATGACTAGCAAAGGAGAAGAACTTTTCAC** and **AAAAAAAAGCTTGCTGAGGTTTAATTAATTATTTGTATAGTTGATCCATGCC** and cloning the PCR product into pET28a via the BamHI and HindIII restriction sites. The coding sequence of iridoid synthase lacking 30 amino acids N-terminally was amplified from pDEST17-*ISY* [see reference 2 from main text] using primers GGCCTTAAUTGAAAACCTGTATTTTCAGGGCGTGGCACTAGTAGTAGGAGTCACC **and** GGTTTAAUCTAAGGAATAAACCTATAATCCCTCATCTTATCAAT and USER-cloned into Nt.BbvCI/PacI-digested pET28au [Nour-Eldin et al. (2010) *Methods Mol Biol* **35**:e55]. The tagged, truncated protein was expressed from the resulting plasmid and purified by

affinity purification as described in reference 2 of the main text. Apart from six consecutive His residues, the N-terminal protein tag (MGSSHHHHHHSSGLVPRGSHMASMTGGQQMGRGSAEALIENLYFQG) also provided a TEV cleavage site (NLYFQG), but all attempts to remove the tag using TEV protease were unsuccessful.

Amino acid sequence of iridoid synthase. Residues crossed out were deleted in the truncated construct.:
~~mswwwkrsigagknlpnqnkengvcksyks~~valvvgvtgivgsslaevlklpdtpggpwkvygvarrpcpvwlakkpveyiqc
dvsdnqetisklsplkdithifyvswigsedcqtnatmfknilnsvipnasnlqhvclqtgikhyfgifeegskvvphdspft
edlprlnvpnfyhdledilyeetgknnltwsvhrpalvfgfspcsmmnivstlcvyatickhenkalvypgsknswncyadav
dadlvaeheiwaavdpkaknqvlncnngdvfkwkhiwkklaeefgiemvgyvegkeqvslaelmkdkdqvwdeivkknnlvpt
klkeiaafwfadiafcsenlissmnkskelgflgfrnsmksfvscidkmrdyrfip

TLC- and GC–MS-based assays (small-scale)

Reactions (200 µL) were set up in glass vials using 200 µM substrate, 600 µM NADPH, 20 mM MOPS pH 7.0, and 0.5 µg of purified protein, and were terminated after 1 h by adding 250 µL CH_2Cl_2. The organic phase was used directly for GC–MS analysis. For analysis by TLC, 150 µL of the organic phase was vacuum-concentrated to approximately 10 µL, spotted onto normal-phase TLC plates, run using 10:1 hexanes: ethyl acetate, and visualized with anisaldehyde stain. For the reaction time course, an analogous 1mL reaction containing 500 µM 8-oxogeranial, 1.5 mM NADPH, 20 mM MOPS pH 7.0, and 180 ng of purified protein was set up. The reaction was terminated at different times by adding 100 µL of ethyl acetate to 50-µL aliquots, and the organic phase was used directly for GC–MS analysis.

Milligram-scale (large-scale) assays

The milligram-scale enzyme assays were carried out using an NADPH-generation/regeneration system consisting of glucose-6-phosphate (G6P), glucose-6-phosphate dehydrogenase (G6PDH) and $NADP^+$.

a) Enzymatic synthesis of (E)-9,9-difluoro-3,7-dimethylnona-6,8-dienal **12**

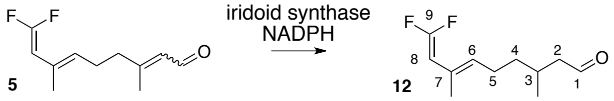

The reaction of 8-(difluoromethylene)geranial **5** with the iridoid synthase was set up in 375 mL of 50 mM MOPS pH 7.0 using 937 µg of purified enzyme, 15 mg of 8-(difluoromethylene)geranial **5** (0.0750 mmol), 110 mg of G6P, 250 U of G6PDH and 17.0 mg of $NADP^+$. The reaction was monitored continuously by TLC using a short-wave ultraviolet lamp for detection, and went to completion after 1 h. The products were extracted with dichloromethane (3 × 200 mL), 1 h later. The combined organic layers were washed with H_2O (200 mL), dried over sodium sulfate, filtered and concentrated *in vacuo* afforded compound **12** as light yellow oil (14.9 mg, 0.0737 mmol, 98%).

^{1}H NMR (400 MHz, C_6D_6): δ = 9.32 (t, ^3J = 1.9 Hz, 1 H, 1-H), 5.64 (dd, $^3J_{HF}$ = 5.1, 26.9 Hz, 1 H, 8-H), 5.06 (t, ^3J = 7.2 Hz, 1 H, 6-H), 1.84–1.66 (m, 5 H, 2-H, 3-H, 5-H), 1.66–1.62 (m, 3 H, 7-CH_3), 1.10–0.83 (m, 2 H, 4-H), 0.68 (d, ^3J = 6.4 Hz, 3 H, 3-CH_3) ppm. **^{13}C NMR** (100 MHz, C_6D_6): δ = 200.6 (+, C-1), 155.5 (C_{quart}, dd, $^1J_{CF}$ = 285.2, 297.2 Hz, C-9), 130.8 (+, dd, $^4J_{CF}$ = 8.8, 5.4 Hz, C-6), 128.7 (C_{quart}, C-7), 86.4 (+, dd, $^3J_{CF}$ = 27.2, 12.5 Hz, C-8), 50.8 (–, C-2), 36.5 (–, C-4), 27.6 (+, C-3) 25.5 (–, C-5), 19.7 (+, 3-CH_3), 14.5 (+, d, $^4J_{CF}$ = 5.9 Hz, 7-CH_3) ppm. **^{19}F NMR** (376 MHz, C_6D_6): δ = –85.83 (dd,

$^2J_{FF}$ = 42.7 Hz, $^3J_{FH}$ = 26.7 Hz, 1 F, *cis*-F), –85.24 (dd, $^2J_{FF}$ = 42.7 Hz, $^3J_{FH}$ = 5.00 Hz, 1 F, *trans*-F) ppm. See selected spectra in Supplementary Figure 3. HR-GCMS calculated for $C_{11}H_{16}F_2O$ $[M]^+$: 202.1169, found 202.1163.

b) Enzymatic synthesis of (1R,2S,5S)-2-methyl-5-(prop-1-en-2-yl)cyclopentanecarbaldehyde **11**

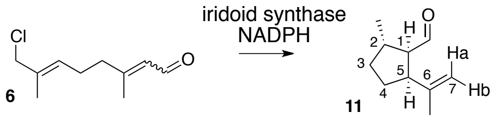

The reaction of 8-chlorogeranial **6** with the iridoid synthase was set up in 290 mL of 50 mM MOPS pH 7.0 using 722 µg of purified enzyme, 10.8 mg of 8-chlorogeranial **6** (0.0581 mmol), 85.5 mg of G6P, 250 U of G6PDH and 13.1 mg of $NADP^+$. The reaction was monitored continuously by TLC using a short-wave ultraviolet lamp for detection, and went to completion after 1 h. The products were extracted with dichloromethane (3 × 200 mL), 1 h later. The combined organic layers were washed with H_2O (200 mL), dried over sodium sulfate, filtered and concentrated under reduced pressure Due to the fact, that the product is probably volatile, the pressure was kept over 600 mbar. The crude product (11.5 mg) was purified *via* chromatography on silica (petrol ether/Et_2O = 20/1) affording compound **11** as colorless oil in about 57% yield (5 mg, 0.0329 mmol).

^{1}H NMR (400 MHz, CD_2Cl_2): δ = 9.48 (d, ^3J = 3.8 Hz, 1 H, CHO), 4.85–4.82 (m, 1 H, 7-H_a), 4.81–4.79 (m, 1 H, 7-H_b), 2.92–2.82 (m, 1 H, 5-H), 2.42–2.38 (m, 1 H, 1-H), 2.37–2.30 (m, 1 H, 2-H), 2.08–1.99 (m, 1 H, 3-H_a), 1.88–1.80 (m, 1 H, 4-H_a), 1.75–1.73 (m, 3 H, 6-CH_3), 1.73–1.71 (m, 1 H, 4-H_b), 1.28–1.26 (m, 1 H, 3-H_b), 1.04 (d, ^3J = 6.7 Hz, 3 H, 2-CH_3) ppm. **^{13}C NMR** (100 MHz, CD_2Cl_2): δ = 204.2 (+, CHO), 144.7 (C_{quart}, C-6), 111.5 (–, C-7), 60.8 (+, C-1), 49.3 (+, C-5), 34.8 (+, C-2), 34.5 (–, C-3), 30.7 (–, C-4), 23.2 (+, 6-CH_3), 20.8 (+, 2-CH_3) ppm. The signal for 3-H_b was overlapped by solvent signals. Since the product is volatile, the solvent could not be totally evaporated. However, HSQC and COSY showed coupling at this position. NOESY showed coupling between 1-H and 5-H as well as between 1-H and 2-CH_3. See selected spectra in Supplementary Figure 4. HR-GCMS calculated for $C_{10}H_{16}O$ $[M]^+$: 152.1201, found 152.1197.

It should be mentioned that the molecule **6** decomposes in aqueous solution within approximately 3 hours. However, time courses showed that the enzymatic reaction is complete within several minutes. Since the enzymatic reaction is so much faster than the decomposition, mechanistic studies were not affected.

Spectrophotometry-based assays (kinetic and substrate specificity studies)

For kinetic studies, the absorbance at 340 nm of 200 µL assays was measured using a 96-well plate reader. See the legend to Supplementary Fig.5 for further experimental details. Data were collected for 3–15 min, with individual measurements being taken for a single sample every 10 s when using full plate. The measured absorbances were plotted manually on Excel and the initial delta absorption/delta time values were calculated for the linear part of each reaction. NADPH consumption rates were calculated from these delta absorption/delta time values considering background NADPH decay and an extinction-coefficient-like value (dependent on assay volume) calculated separately. The Michaelis-Menten curve was fitted using Origin software.
